# Lymph node macrophage-targeted interferon alpha boosts anticancer immune responses by regulating CD169-positive phenotype of macrophages

**DOI:** 10.1186/s12943-025-02324-8

**Published:** 2025-05-03

**Authors:** Ryo Fukuda, Yukio Fujiwara, Hitoshi Maeda, Cheng Pan, Yuki Minayoshi, Hiromu Yano, Yuki Mizuta, Mei Takano, Rin Yamada, Yoichi Saito, Kenshiro Hirata, Shuhei Imoto, Keishi Yamasaki, Kentaro Oniki, Junji Saruwatari, Masaki Otagiri, Hiroshi Watanabe, Yoshihiro Komohara, Toru Maruyama

**Affiliations:** 1 Department of Biopharmaceutics, Graduate School of Pharmaceutical Sciences, Kumamoto University, 5-1 Oe-honmachi, Chuo-ku, Kumamoto, 862-0973 Japan; 2 Department of Cell Pathology, Graduate School of Medical Sciences, Faculty of Life Sciences, Kumamoto University, 1-1-1 Honjo, Chuo-ku, Kumamoto, 860-8556 Japan; 3 Laboratory of Biopharmaceutics, Kyoto Pharmaceutical University 5 Nakauchi-cho, Misasagi, Yamashina-ku, Kyoto 607-8414, Japan; 4 Department of Tumor Pathology, Graduate School of Health Sciences, Faculty of Life Sciences, Kumamoto University, 4-24-1 Honjo, Chuo-ku, Kumamoto, 862-0976 Japan; 5 Laboratory of Bioengineering, Faculty of Advanced Science and Technology, Kumamoto University, 2-39-1 Kurokami, Chuo-ku, Kumamoto, 860-8555 Japan; 6 Department of Pharmaceutical Sciences, Faculty of Pharmaceutical Sciences, Sojo University, 4-22-1 Ikeda, Kumamoto, 860-0082 Japan; 7 Division of Pharmacology and Therapeutics, Graduate School of Pharmaceutical Sciences, Kumamoto University, 5-1 Oe-honmachi, Chuo-ku, Kumamoto, 862-0973 Japan

**Keywords:** Lymph node, Macrophage, CD169, Albumin, IFNα, Drug delivery system, Cancer immunotherapy, Targeted therapy

## Abstract

**Background:**

CD169^+^ macrophages in lymph nodes (LNs) activate cytotoxic T lymphocytes (CTLs), which play a crucial role in anticancer immunity, through antigen presentation and co-stimulation by CD169. Interferon alpha (IFNα) is capable of inducing the CD169^+^ phenotype of macrophages; however, its clinical applications have been hindered by pharmacokinetic limitations—low LN distribution and an inability to target macrophages. To overcome these issues, this study genetically fused mouse IFNα (mIFNα) with mannosylated mouse serum albumin (Man-MSA), and investigated the antitumor effects of the hybrid protein (Man-MSA-mIFNα) or its add-on effects with programmed death-ligand 1 (PD-L1) blockade.

**Methods:**

To confirm the possibility of CD169^+^ macrophage-mediated T cell priming, positional information about individual immune cells in LNs of cancer patients was obtained. Traits of Man-MSA-mIFNα, which was prepared using *Pichia pastoris* to form the high-mannose structure, were characterized by several physicochemical methods. To evaluate the lymphatic drainage of Man-MSA-mIFNα, radioiodine or Cy5-labeled Man-MSA-mIFNα was subcutaneously administered in mice, and then the radioactivity or fluorescence in LNs was analyzed. CD169-diphtheria toxin (DT) receptor (CD169-DTR) mice in which LN CD169^+^ macrophages can be depleted by DT injection were used to verify whether the antitumor effect of Man-MSA-mIFNα is dependent on LN CD169^+^ macrophages.

**Results:**

Multiplex tissue imaging predicted close proximity of CD169^+^ macrophages and T cells and positive correlation between the number of CD169^+^ macrophages and T cells in neighborhoods in LNs of cancer patients. Physicochemical analyses showed that Man-MSA-mIFNα was formed from the fusion of the intact Man-MSA and mIFNα. Man-MSA-mIFNα efficiently induced the CD169^+^ phenotype of macrophages by its high LN distribution and macrophage-targeting capability, and significantly exerted antitumor activity through CD8^+^ T cell activation in the LNs, whereas its antitumor effects were canceled in CD169-DTR mice. Finally, combination therapy with PD-L1 blockade markedly suppressed tumor growth in MB49-bearing mice, which exhibit resistance to PD-L1 blockade monotherapy.

**Conclusions:**

The present study successfully designed and developed Man-MSA-mIFNα, which efficiently induces the CD169^+^ phenotype in LN macrophages, contributing to the antitumor immunity. The findings suggest that our novel strategy targeting CD169⁺ macrophages could be a promising immunotherapy for cancer patients who are unresponsive to immune checkpoint inhibitors.

**Graphical Abstract:**

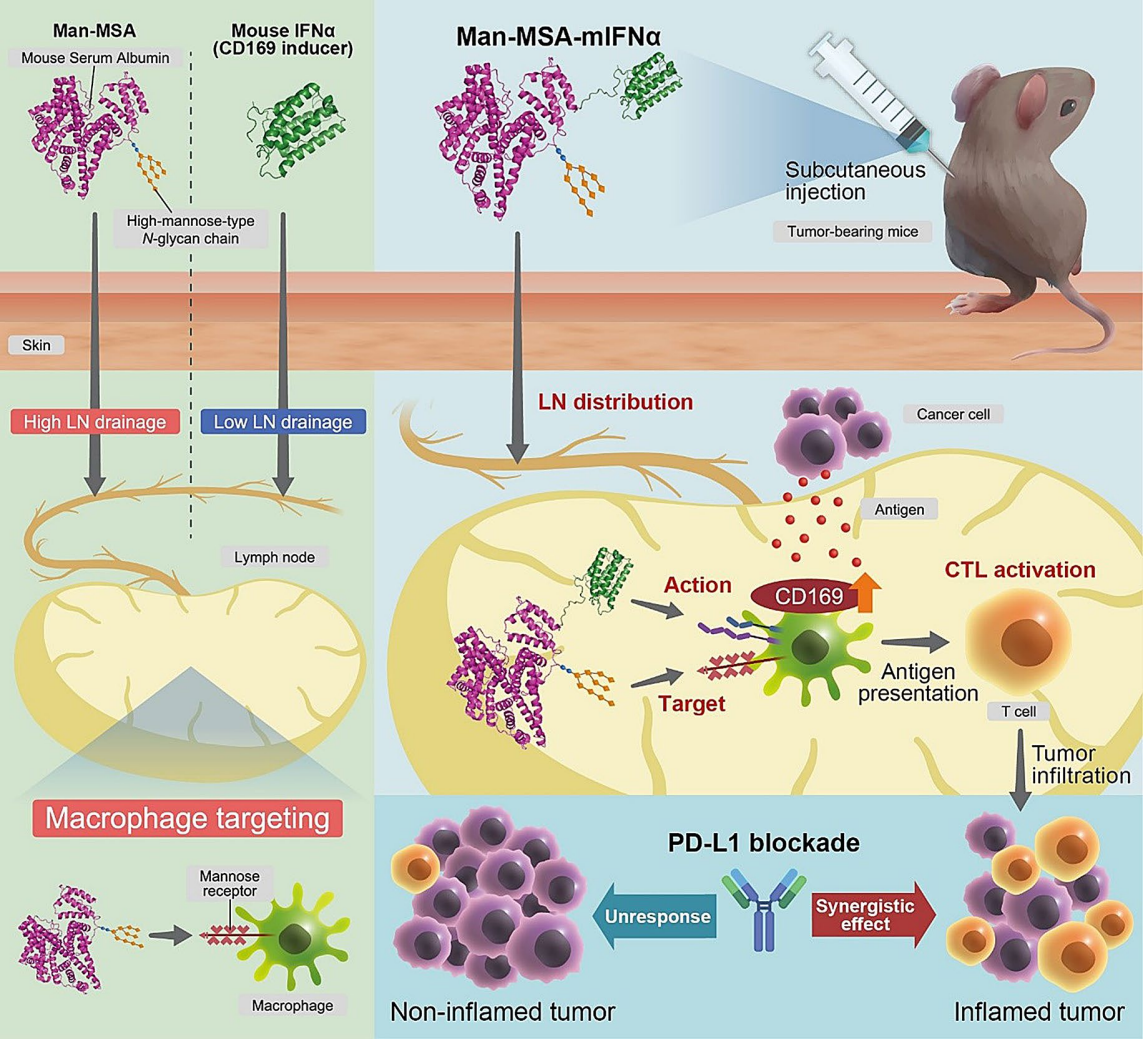

**Supplementary Information:**

The online version contains supplementary material available at 10.1186/s12943-025-02324-8.

## Background

Tumor immunotherapy enhances the body’s natural immune system to combat cancer. This type of treatment leverages innate immunity, including macrophages and dendritic cells, and acquired immunity, encompassing T and B cells. Activated cytotoxic T lymphocytes (CTLs) play a crucial role in cancer cell destruction by releasing inflammatory cytokines such as interferon-gamma (IFNγ), perforin, and granzymes. Studies show that an increased presence of CTLs in tumors is linked to improved patient outcomes [1]. Extensive research has been done on molecules and applications that stimulate CTLs. One established method involves immune checkpoint inhibitors that activate CTLs by targeting immune checkpoint molecules on the surfaces of cancer and immune cells, including macrophages. The use of antiprogrammed death-1/ligand-1 (anti-PD-1/PD-L1) antibodies is approved for treating various cancers, such as malignant melanoma, non-small cell lung cancer, and renal cell carcinoma [2]. Drugs targeting other immune checkpoint molecules are also available and have proven effective in cancer treatment. Ongoing research in this field aims to refine methods for CTL activation to enhance the efficacy of cancer immunotherapy.

Understanding the mechanisms underlying CTL activation is crucial for developing cancer immunotherapy methods. Mellman et al. synthesized their insights into the CTL induction process and developed the concept of the “cancer-immunity cycle [3].” According to this model, dendritic cells play a significant role in CTL activation. However, Asano et al. uncovered findings that diverge from this theory. Their research involved administering dead cancer cells to mice and monitoring their movement within the lymphatic system. They reported that CD169^+^ macrophages, not dendritic cells, were mainly responsible for taking up cancer cells. This process facilitated CTL activation via antigen presentation [4]. Moreover, Zhang et al. and Xiong et al. discovered that CD169 is a vital co-stimulatory molecule for activating CTLs in vitro [5, 6]. Based on these findings, several clinical studies have validated the role of CD169^+^ macrophages in cancer patients. Ohnishi et al., for example, identified a strong correlation between the number of CD169^+^ macrophages in lymph nodes (LNs) of colorectal cancer patients, the number of CTLs in tumor tissues, and the patients’ prognoses [7]. Comparable results have emerged from research on other types of cancer [8–13], underscoring the potential of effectively inducing CD169^+^ macrophages in vivo as a promising strategy for stimulating CTLs.

Several biomolecules capable of inducing CD169 expression have been identified, with interferon-alpha (IFNα) standing out due to its efficacy, mediated by the activation of signal transducer and activator of transcription (STAT) 1 and 2 [7, 13, 14]. Recent cancer immunotherapy targeting LNs has been focused on delivering stimulator of interferon genes (STING) agonist or Toll-like receptor 9 (TLR9) agonist to antigen-presenting cells for production of IFNα [15, 16]. However, the strategy to deliver IFNα directly to LN macrophages to induce CD169 more efficiently has not yet been realized. There are two pharmacokinetic challenges based on the molecular characteristics of IFNα that must be overcome to establish an effective IFNα delivery system to LN macrophages. The first challenge is the low LN drainage of IFNα, attributed to its molecular size of approximately 4.4 nm [17]. Generally, molecules sized between 5 nm and 100 nm can drain into lymphatic capillaries after subcutaneous injection [18], but the smaller size of IFNα may hinder its ability to enter these capillaries effectively. The second issue pertains to the targeting efficiency of IFNα to macrophages. The widespread expression of type I IFN receptors on various cells necessitates an elevated dose of IFNα to achieve the desired therapeutic effect on LN macrophages. However, increasing the dosage also elevates the risk of side effects, adversely affecting the patient’s quality of life and escalating medical costs. Therefore, drug delivery nanotechnology is essential for harnessing IFNα’s potential in cancer immunotherapy, especially in inducing the CD169^+^ phenotype in LN macrophages.

Human serum albumin (HSA), the most plentiful protein in human blood, is distributed within intravascular and extravascular spaces. Approximately 40% of total HSA is found intravascularly while the remaining 60% is located in the extravascular space, which includes the interstitial and lymphatic fluids. Owing to its molecular size of approximately 10 nm, HSA efficiently drains into LNs after subcutaneous administration. This property has led researchers to explore the potential use of HSA as a diagnostic tool for detecting LN metastasis [19]. These insights have led us to propose a novel approach to overcoming the initial pharmacokinetic challenge associated with low LN drainage of IFNα by its conjugation with HSA. This hybrid would enhance the lymphatic delivery of IFNα due to the increased molecular size, overcoming a key barrier to utilizing IFNα effectively in cancer immunotherapy.

Wild-type HSA is a simple protein without glycan chains. To deliver drugs to macrophages, we had previously developed a mannosylated HSA (Man-HSA) attached with three mannosyl chains using the *Pichia pastoris* expression system [20]. We also established that Man-HSA and its polyethylene glycol derivative were preferentially distributed to Kupffer cells or tumor-associated macrophages, respectively, via the mannose receptors [21, 22]. These findings clarify that Man-HSA is a suitable carrier for targeting LN macrophages.

In the present study, we proposed a novel strategy of cancer immunotherapy, that is, “induction of the CD169^+^ phenotype in LN macrophages” based on the active targeting of IFNα to LN macrophages. For this purpose, we created the hybrid protein of mouse serum albumin with a highly mannosylated sugar chain (Man-MSA) and mouse IFNα (mIFNα) and subsequently clarified its potential as an “immune booster.” First, to confirm whether the CD169^+^ macrophage was a suitable target for cancer immunotherapy, whole-tissue imaging was conducted at single-cell resolution using LN samples from colorectal carcinoma patients and tumor-bearing mice. Then, Man-MSA-mIFNα was developed by albumin fusion technology. We thoroughly investigated its physicochemical and pharmacokinetic characteristics and assessed its capacity to induce CD169 expression both in vitro and in vivo. Moreover, the effects of Man-MSA-mIFNα on tumor growth and antitumor immunity were evaluated using several types of tumor-bearing mice. Finally, we also examined the combinational effect with an immune checkpoint inhibitor on tumor development. This study has the potential to provide the personalized medicine that tailors cancer immunotherapy based on the CD169^+^ macrophage content in LNs.

## Methods

### Multiplex tissue imaging and analysis

The procedure is similar to a previously described method [23]. Briefly, a formalin-fixed, paraffin-embedded block was sliced into 3 μm-thick sections, and a tissue section was mounted on a glass coverslip, deparaffinized by heating at 60 °C for 20 min, and then submerging in xylene. The tissue was then rehydrated in a graded ethanol series. The sections were immersed in citrate buffer solution (10 mm, pH 6.0) and heated in a pressure cooker for antigen retrieval. After cooling to room temperature, the tissue was stained with antibodies for 3 h at room temperature in a humidity chamber. After fixation with a post-staining fixing solution for 10 min, 100% methanol for 5 min, and final fixative solution for 20 min, the tissue was stored in PBS at 4 ℃ until image acquisition. Multiplex imaging was executed using a microfluidics instrument (Akoya Biosciences, Menlo Park, CA), a fluorescence microscope (BZ-X700; Keyence, Osaka, Japan), and CODEX Instrument Manager software (Akoya Biosciences). An automated microfluidics system performed iterative annealing and stripping of fluorophore-labeled oligonucleotide barcodes complementary to the barcodes attached to antibodies. The raw image files were processed using CODEX processor version 1.8 (Akoya Biosciences). The antibodies and fluorophore-labeled oligonucleotide barcodes are described in Table S1.

QPTIFF files were imported to QuPath version 0.3.2. We extracted six 500 μm × 500 μm square regions in the image and performed cell segmentation on the basis of the nuclear stain using the cell detection algorithm in the software. To classify cells as being positive or negative for each marker, we generated training data for cell classification by deciding the positive and negative categories for every 10–30 cells in the regions based on fluorescence intensity. The results comprising the position (X, Y) and fluorescence intensity of each cell were exported as a csv file.

The csv file was imported to CytoMAP version 1.4.21. A detailed description of the workflow and functions built into CytoMAP is available in the online user manual. The representative area was subdivided into four regions by a 50 μm-radius raster-scanned neighborhoods function, which uses the Davies Bouldin Score and self-organizing map clustering methods, based on each fluorescence intensity. The analyses for neighborhood composition and Pearson correlation coefficients were performed based on the fluorescence intensity using the “Region Heatmaps” and “Cell-Cell Correlation” functions.

### Multiplex immunohistochemistry

 Rabbit monoclonal antibodies (CD34, CD8, CD4), a rabbit polyclonal antibody (CD169), and a rat monoclonal antibody (B220) were used. Immunoreactions were visualized with aminoethyl carbazole (NICHIREI BIOSCIENCES INC., Tokyo, Japan), following a previously described protocol [24]. Slides were scanned using a NanoZoomer S20 scanner, then sequentially destained, stripped, and restained as described [24]. Color deconvolution and image fusion were performed with HALO to create multichannel pseudo-fluorescent images. For triple staining of lymph nodes, anti-IFNAR1, anti-CD68, and anti-CD206 antibodies were used. Details of antibodies are listed in Table S1.

### Preparation of fusion proteins

The designed fusion proteins are composed of MSA or MSA (D494N) linked to mIFNα2 (N78Q) via a polypeptide linker (-(Gly-Gly-Gly-Gly-Ser)_2_-). In this study, we replaced asparagine at position 78 of mIFNα2 with glutamine (N78Q) to remove a glycosylation sequence originally present in mIFNα2. The flow chart describing the creation of the MSA (D494N)-mIFNα2 (N78Q) gene using pPIC9 is shown in Fig. S1. PCR was performed using a PfuTurbo DNA polymerase. To isolate the DNA fragment of the base sequence coding for MSA, restriction enzyme Xho1 and Ava1 recognition regions were inserted into the 5′ and 3′ terminals, respectively. Similarly, restriction enzyme Ava1 and EcoR1 recognition regions were inserted into the 5′ and 3′ terminals of the DNA sequence encoding mIFNα2. Then, pPIC9 was digested with Xho1 and EcoR1, and the appropriate pPIC9 fragment was extracted by agarose gel electrophoresis. To obtain the cDNA construct coding for MSA-mIFNα2, DNA fragments (pPIC9, MSA and mIFNα2) were ligated overnight at 16 °C using a DNA ligation kit (Takara bio, Shiga, Japan). The subsequent mutation of MSA (D494N) and mIFNα2 (N78Q) was performed using a Quick Change kit (Takara bio) with the mutagenic primers described in Table S2. Hexa-His-tag was inserted into the C-terminal of mIFNα2 (N78Q), and *Pichia pastoris* (SMD1168 strain) was transformed with pPIC9-MSA (D494N)-mIFNα2 (N78Q) by electroporation according to the manual.

### Pharmacokinetic analysis of ^125^I-labeled proteins

The proteins were radiolabeled with ^125^I according to previously reported procedures [25]. ^125^I-labeled Man-MSA-mIFNα and MSA-mIFNα (1 mg protein kg^− 1^; approximately 7 × 10^6^ counts per minute [CPM] per mouse), MSA (1 mg protein kg^− 1^; approximately 6 × 10^6^ CPM per mouse), and mIFNα (1 µg protein kg^− 1^; approximately 8 × 10^5^ CPM per mouse) were subcutaneously administered into the inguinal region of ICR mice. Inguinal LNs were isolated at each time point after the administration of the ^125^I-labeled proteins, and the radioactivity was measured using an autowell gamma counter (AccuFlex γ7001; Hitachi Aloka Medical, Tokyo, Japan) according to the manufacturer’s instructions.

### Preparation of Cy5-labeled proteins

The lyophilized proteins were dissolved in deionized water to 20 mg mL^− 1^. Cy5 NHS ester (0.5 mg) was added to the protein solution (1 mL), and the solution was mixed by rotation for 1 h at room temperature. The unreacted Cy5 NHS ester was removed by ultrafiltration using an Amicon Ultra Ultracel-PL 10 kDa membrane filter (Merck Millipore, Burlington, MA).

### Pharmacokinetic analysis of Cy5-labeled proteins

To evaluate the LN macrophage-targeting ability of Man-MSA-mIFNα, Cy5-labeled fusion proteins (60 nmol kg^− 1^ for immunofluorescence staining and 6 nmol kg^− 1^ for flow cytometric analysis) were subcutaneously administered into the inguinal region of ICR mice, and the inguinal LNs were isolated 1 h after administration.

### Flow cytometry

LNs or tumors were harvested, minced finely with scissors on ice, and then incubated with collagenase D (Sigma-Aldrich) or Dri tumor & tissue dissociation reagent (Becton Dickinson, NJ) at 37 °C for 30 min. After passing the digested mixture through a 70 μm filter, the cells were incubated with ACK lysis buffer (NH₄Cl [0.15 m], KHCO₃ [10 mm], and EDTA [0.1 mm]) to remove red blood cells and suspended in FACS buffer (EDTA/PBS [2 mm] containing 0.5% FBS). The cells were incubated with FcR blocking reagent (1:50, Miltenyi Biotec, 130-092-575) and Fixable Viability Dye eFluor 780 (1:5,000, Invitrogen, 65-0865-14) at room temperature for 5 min and then stained with antibodies for cell surface markers at 4 °C for 15 min. After washing with FACS buffer, the cells were fixed, permeabilized, and incubated with antibodies for intracellular markers at 4 °C for 30 min. The cells were analyzed on a FACSVerse (Becton Dickinson) flow cytometer with FACSuite (Becton Dickinson) software. The data were analyzed using FlowJo software (Becton Dickinson). The gating strategies are shown in Fig. S2 and Fig. S3, and the antibodies in Table S1.

### Evaluation of CD169 expression in vitro

J774.1 cells were seeded at a density of 3.0 × 10^5^ cells per well in 12-well plates and incubated overnight at 37 °C before the addition of mIFNα (1 IU mL^− 1^), MSA, MSA-mIFNα, or Man-MSA-mIFNα (70 nm) to the cells. The cells were collected after incubation for 24 h at 37 °C, and CD169 expression was evaluated by flow cytometry.

### Evaluation of CD169 expression in vivo

We first assessed the optimal dose of Man-MSA-mIFNα and the time course of CD169 expression. As shown in Fig. S4 and Fig. S5, we confirmed that, 72 h after administration, Man-MSA-mIFNα (0.6 nmol kg^− 1^) showed high CD169 expression on LN macrophages. Next, to compare the in vivo CD169 induction levels of mIFNα and Man-MSA-mIFNα, we evaluated the titer of mIFNα using J774.1 cells and found the induction level of CD169 after treatment with mIFNα (1 IU mL^− 1^) to be equivalent to that after Man-MSA-mIFNα (6 µg mL^− 1^, 0.07 nmol mL^− 1^) (Fig. S6). Thus, mIFNα (8 IU kg^− 1^), MSA-mIFNα (0.6 nmol kg^− 1^), or Man-MSA-mIFNα (0.6 nmol kg^− 1^) was subcutaneously administered into the inguinal region of C57BL/6 N mice. Seventy-two hours after administration, inguinal LNs were harvested, and the CD169 expression on macrophages evaluated by flow cytometry.

### Antitumor effects of Man-MSA-mIFNα

We first evaluated the optimal dosing interval using MB49-bearing mice and found that the administration of Man-MSA-mIFNα once every 4 days, but not once every 7 days, exerted significant antitumor effects (Fig. S7). Therefore, mIFNα (8 IU kg^− 1^), MSA-mIFNα, and Man-MSA-mIFNα (0.6 nmol kg^− 1^) were injected subcutaneously near the tumor once every 4 days. For the in vivo CD4/CD8 depletion experiments, specific antibodies were injected intraperitoneally (100 µg) once every four days after tumor inoculation. The antibodies are described in Table S1. For combination therapy, the mice were treated intraperitoneally with isotype control antibodies (200 µg; BioLegend, 400667) and anti-PD-L1 antibodies (200 µg; BioLegend, 124329) on the 10th and 13th days after tumor inoculation and then, subcutaneously with Man-MSA-mIFNα (0.3 nmol kg^− 1^) once every 4 days. Overall survival benefit of combination therapy was determined by measuring time to required euthanasia based on tumor size (3000 mm³) in accordance with the experimental animal ethics committee at Kumamoto University (A2021-021). The tumor volume was calculated according to the formula V = 0.4 × a × b^2^, where “a” is the major axis, and “b” is the minor axis of the tumor. Tumor-bearing mice were sacrificed on the 18th day after tumor inoculation.

### Statistical analysis

The experimental data were analyzed using GraphPad Prism 9. The means for two-group data were compared by using an unpaired *t*-test, and the means for more than two groups were compared by one-way ANOVA followed by Tukey’s multiple comparison or Bonferroni’s multiple comparison. The log rank test was used in survival analysis to compare the time to death in two or more independent group. The probability value of *p* < 0.05 was considered to be a statistically significant difference.

## Results

### Spatial identification of LN cells in cancer patients and tumor-bearing mice

**Fig. 1:**
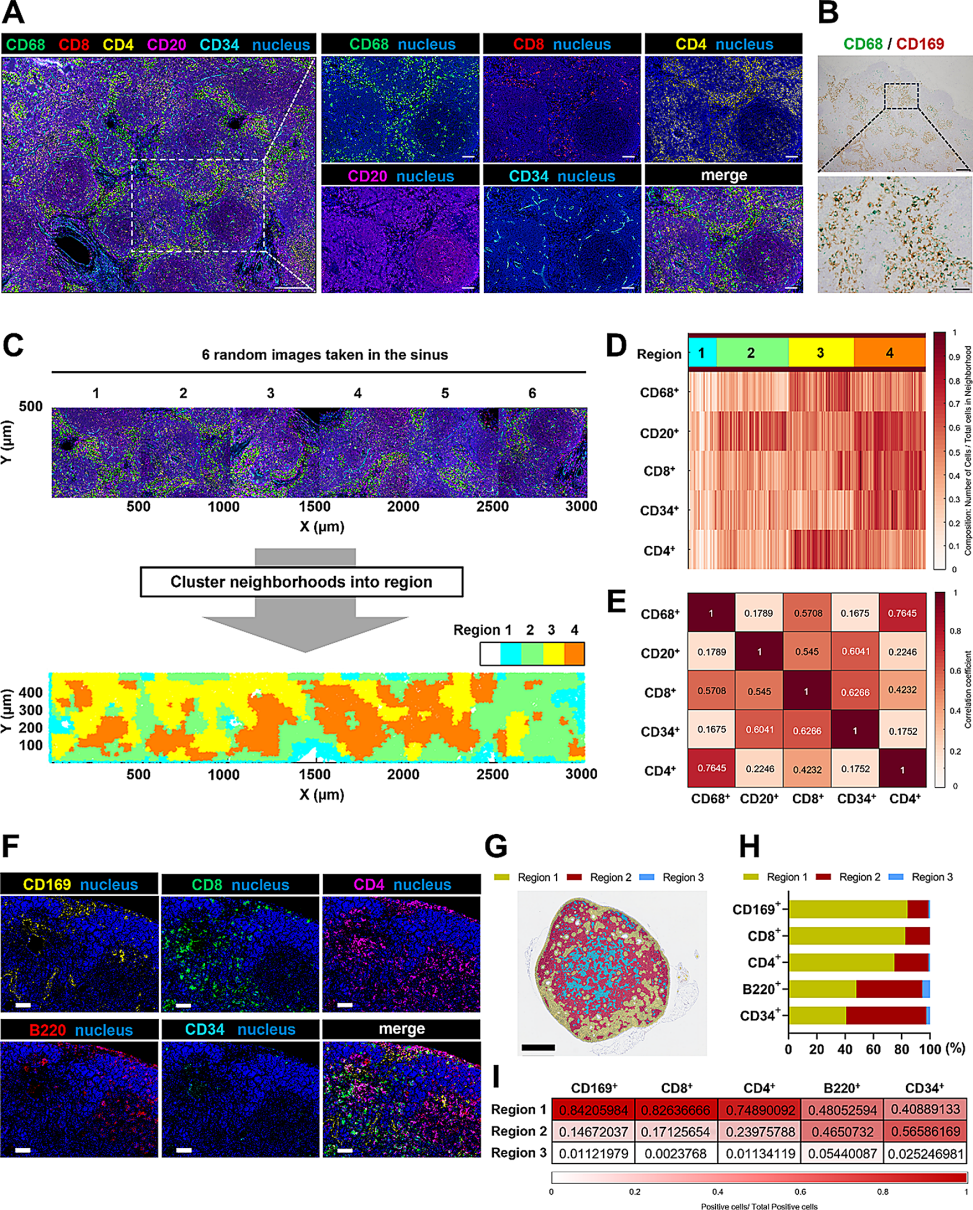
Spatial analysis of regional LN cells in cancer patients and tumor-bearing mice. **A**) Multiple immunostaining for regional LNs of colorectal cancer patients. Scale bar, left: 200 μm, magnified images: 50 μm. **B**) Regional LNs of colorectal cancer patients were stained with CD68 (green) and CD169 (brown). The representative sinus area in the LN (upper, scale bar: 200 μm) was magnified (lower, scale bar: 50 μm). **C**) Representative 2D (X, Y) tissue images (500 × 500 μm) in the sinus (upper) and positional plot of the neighborhoods color coded by the region type (lower). **D**) Heatmap of the neighborhood composition (the number of cells per total cells in neighborhood). **E**) Heatmap of the Pearson correlation coefficients of the number of cells per neighborhood from all regions. **F**) Multiple immunostaining for regional LNs of MB49-bearing mice. Scale bar: 50 μm. **G**) Positional plot of the neighborhoods color coded by the region type. Scale bar: 500 μm. **H**) Percentage of each cell in each region. **I**) Heatmap of the number of positive cells in each region per total positive cells in all area. These results are representative of two independent experiments

To confirm the possibility of CD169^+^ macrophage-mediated T cell priming in LNs of cancer patients, we first predicted cell-cell interactions with critical physiological roles at both cellular and tissue levels by obtaining positional information about individual cells (macrophages, CD8^+^ and CD4^+^ T cells, B cells, and vascular endothelial cells). This information was obtained by applying a single-cell and spatial multi-omics system, the Akoya PhenoCycler system [23], to regional LN biopsies of colorectal carcinoma patients. As shown in Fig. 1A, we immunostained a regional LN of a colorectal carcinoma patient with anti-CD68 (macrophage), anti-CD8 (CD8^+^ T cell), anti-CD4 (CD4^+^ T cell), anti-CD20 (B cell), and anti-CD34 (vascular endothelial cell) antibodies and visualized each cell in the LN sinuses, which contain an abundance of CD169^+^ macrophages (Fig. 1B) [7, 26]. We next imported the fluorescence intensity and positional information (X, Y) of individual cells to CytoMAP, a spatial analytics platform that incorporates diverse statistical and visualization modules [27]. The tissue images (500 × 500 μm) were subdivided into 50 μm-radius neighborhoods, which were mapped based on the cellular composition. Four regions with different cellular compositions were defined using the “raster scanned neighborhoods” function (Fig. 1C). We further visualized the mean channel intensities per neighborhood with heatmaps and found that each region has the following cellular characteristics: Region 1, mixed cellular composition (macrophages, CD8^+^ and CD4^+^ T cells, B cells, vascular endothelial cells) with low cell density; Region 2, B cell-rich composition; Region 3, macrophage- and CD8^+^ and CD4^+^ T cell-rich composition; Region 4, mixed cellular composition (macrophages, CD8^+^ and CD4^+^ T cells, B cells, vascular endothelial cells) with high cell density (Fig. 1D and Fig. S8A). In addition, Pearson correlation coefficients of the number of cells per neighborhood showed that the number of macrophages positively correlated with the number of CD8^+^ (*r* = 0.57) and CD4^+^ T cells (*r* = 0.76) in the neighborhoods (Fig. 1E and Fig. S8B). Furthermore, similar results were observed in regional LNs of tumor-bearing mice that had been subcutaneously injected with MB49 bladder carcinoma cells in the inguinal area (Fig. 1F-I). These results indicate that there were regions where CD169^+^ macrophages may prime T cells within the regional LNs of cancer patients and tumor-bearing mice.

## Expression of mannose receptors and type I IFN receptors in LN macrophages

To verify whether subcutaneously administered Man-HSA is distributed to macrophages in the LN sinuses, we first examined CD206 (mannose receptor) expression in the regional LNs of a colorectal carcinoma patient. Combined staining with hematoxylin-eosin (H.E.) and immunohistochemistry with antibodies against CD163 (a marker for LN sinus macrophages) and CD206 identified the sinus [26], which is marked with an asterisk, and found high expression of CD206 and CD163 in subcapsular and medullary LN sinuses (Fig. S9A). Furthermore, triple immunostaining with antibodies for CD68, CD206, and interferon alpha/beta receptor 1 (IFNAR1) showed that macrophages in LN sinuses expressed both CD206 and IFNAR1 (Fig. S9B).

## Lymphatic drainage of albumin in mice

To investigate whether albumin possesses characteristics for lymphatic drainage, we subcutaneously administered radioiodine (^125^I)-labeled MSA or mIFNα to the inguinal region of mice. More than 20% of the initial dose (ID) per tissue (g) of MSA was distributed in inguinal LN an hour after the administration, whereas the distribution was lower in the case of mIFNα (Fig. S10). We also observed a marked reduction in radioactivity in the mIFNα-administration group at 6 h after the administration. Once the ^125^I-labeled proteins are taken up into cells through receptor-mediated endocytosis and degraded in lysosomes, the radio-catabolites are rapidly excreted from the cells [28]. Therefore, mIFNα was supposed to be taken up by the type I IFN receptor-expressing cells and degraded within 6 h after the administration [29]. These results suggest that HSA may improve lymphatic drainage of IFNα. Therefore, we attempted to develop a genetically fused chimeric protein of mIFNα and Man-MSA because the IFN activity, albumin kinetics, and immunogenic response are largely dependent on animal species [30–32].

## Design and physicochemical properties of Man-MSA-mIFNα

**Fig. 2:**
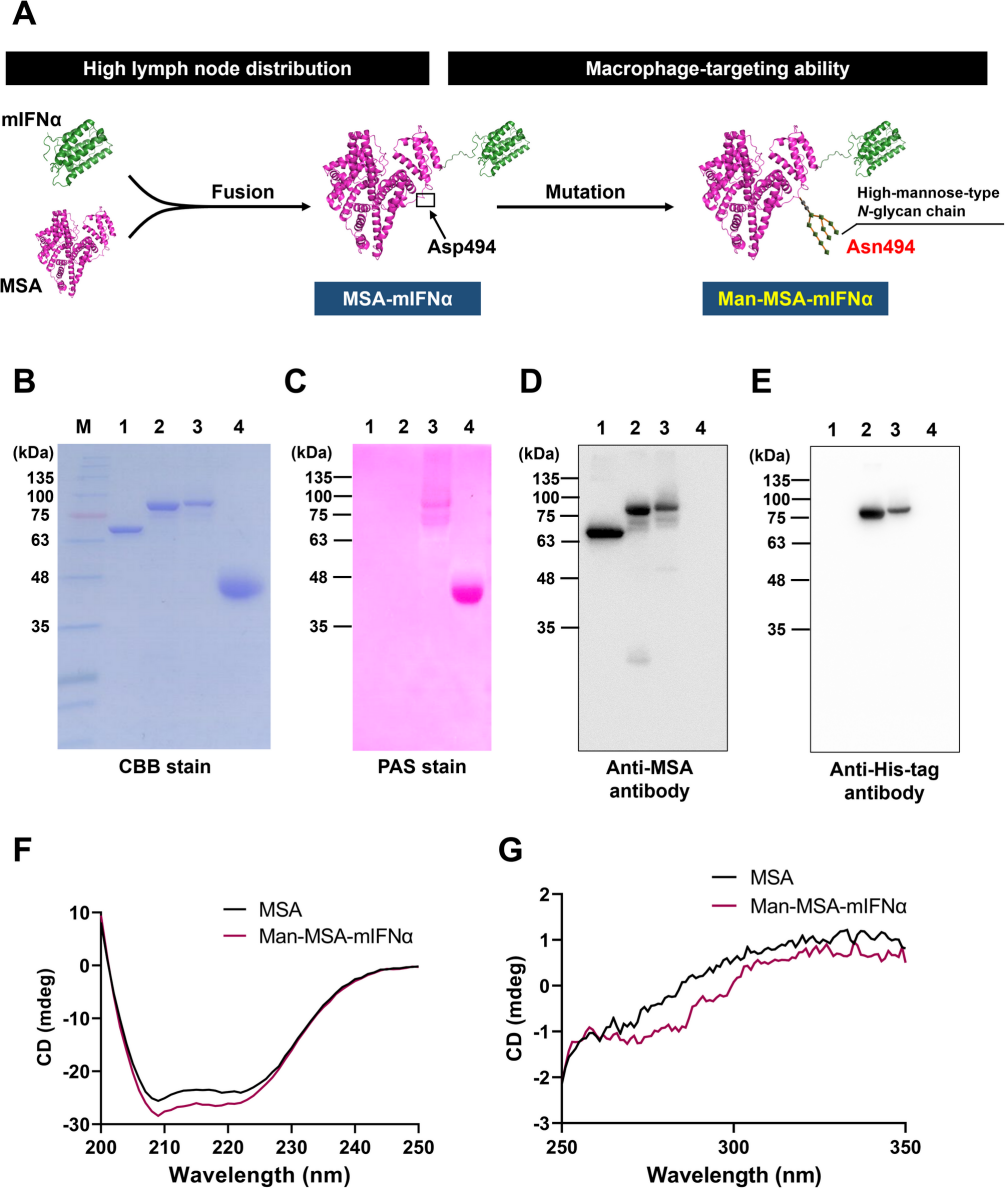
Physicochemical characterization of Man-MSA-mIFNα. **A**) Experimental scheme for the development of Man-MSA-mIFNα. **B**) CBB stain after SDS-PAGE. **C**) PAS stain after SDS-PAGE. **D**,** E**) MSA and His-tag were detected by western blot analysis. M, Molecular weight marker; 1, MSA; 2, MSA-mIFNα; 3, Man-MSA-mIFNα; 4, AGP. **F**) Far-UV and **G**) near-UV spectra were recorded from 200 to 250 nm and from 250 to 350 nm, respectively. These results are representative of three independent experiments

We had previously developed Man-HSA by inserting three *N*-glycation sequences (D63N, A320T, and D494N) into HSA [20]. Analysis using matrix-assisted laser desorption ionization-time of flight-mass spectrometry revealed that the D63N variant had 0–1 mannose residues, A320T had 1–2, and D494N had 11 mannose residues, indicating that D494N was largely responsible for the mannosylation [20]. The clustering effect of highly mannosylated structures plays a crucial role in binding to the mannose receptor [33]. To obtain highly mannosylated forms of the protein while minimizing the mutations, the present study fused His-tag-conjugated mIFNα with mutated MSA (Man-MSA) whose aspartic acid (D) at position 494 was replaced with asparagine (D494N) (Fig. 2A). We first performed sodium dodecyl sulfate-polyacrylamide gel electrophoresis (SDS-PAGE) of the purified fusion proteins to confirm the fusion of mIFNα and mutated MSA. Coomassie Brilliant Blue (CBB) staining showed that the position of the Man-MSA-mIFNα band was slightly higher than that of MSA-mIFNα, but both bands corresponded to the theoretical molecular weight (Fig. 2B). The presence of mannose chains in Man-MSA-mIFNα was further corroborated through Periodic acid-Schiff (PAS) staining. PAS stain reacted positively with Man-MSA-mIFNα and α1-acid glycoprotein (AGP), while such a positive reaction was not observed in the case of MSA-mIFNα (Fig. 2C). Western blotting analyses were carried out using anti-MSA and anti-His-tag antibodies to confirm the presence of MSA and His-tag-conjugated mIFNα in the fusion protein. Man-MSA-mIFNα and MSA-mIFNα reacted positively with both antibodies, but MSA positively reacted with only anti-MSA antibodies (Fig. 2D, E). These data indicate that Man-MSA-mIFNα was formed from the fusion of the intact Man-MSA and mIFNα. We further analyzed the secondary and tertiary structures of Man-MSA-mIFNα by circular dichroism (CD) spectroscopy. As shown in Fig. 2F, G, the CD spectrum of Man-MSA-mIFNα was similar to that of MSA, indicating that Man-MSA largely retained the characteristic structure of MSA despite fusion with mIFNα.

### Intralymph node distribution of Man-MSA-mIFNα and its capacity to induce the CD169^+^ phenotype of macrophages

**Fig. 3:**
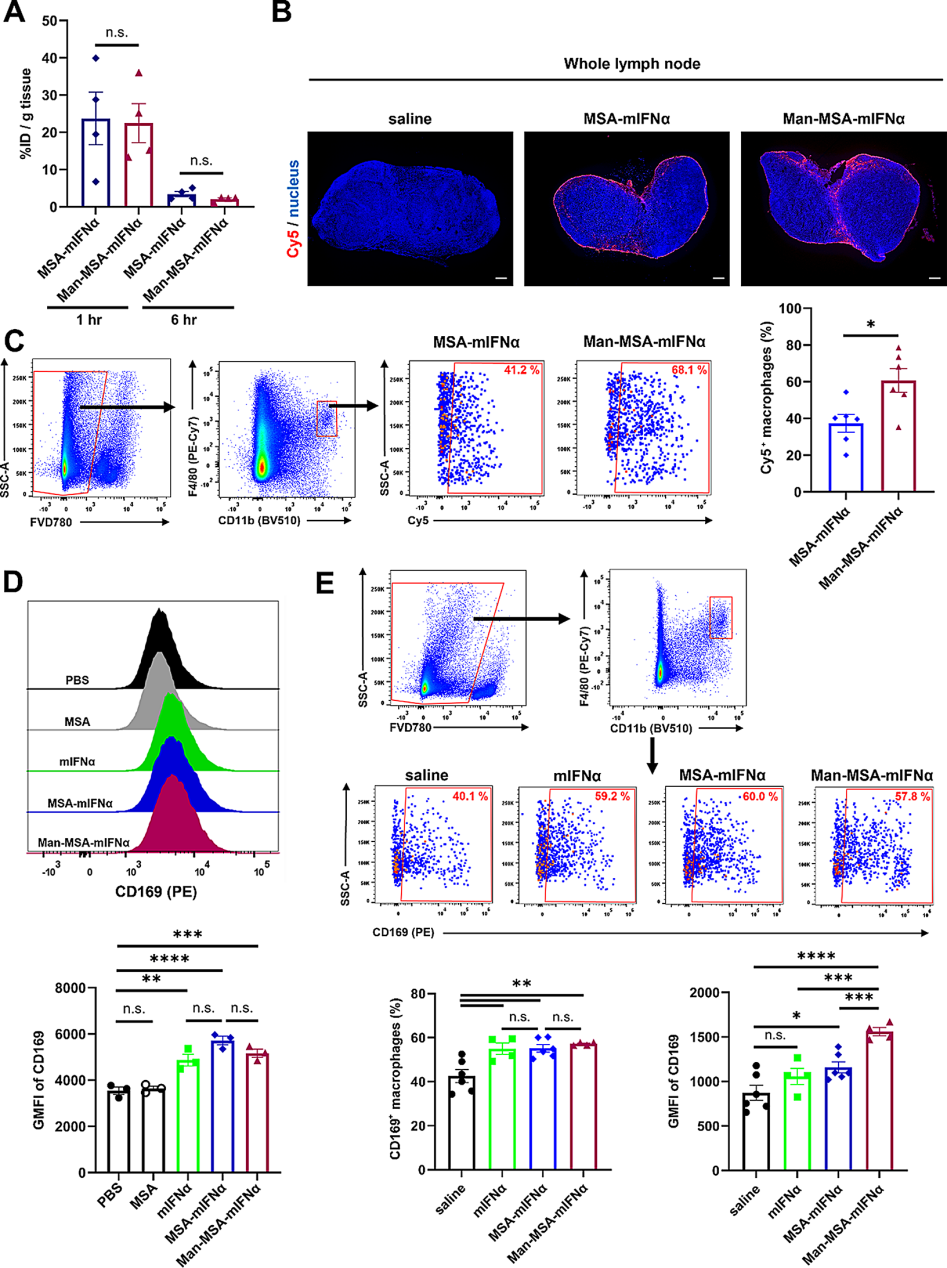
Intralymph node distribution of Man-MSA-mIFNα and its capacity to induce CD169^**+**^ phenotype of macrophages. **A**) LN distribution was measured 1 h and 6 h after administration (s.c.) of each protein (*n* = 4). This experiment was performed once. **B**) Distribution of Cy5-labeled fusion proteins in LNs observed 1 h after subcutaneous administration. Scale bar: 200 μm. **C**) The rate of Cy5-incorporated cells in CD11b^+^ F4/80^+^ macrophages 1 h after subcutaneous administration was evaluated by flow cytometry (*n* = 6). **D**) J774.1 cells were incubated with each protein, followed by the evaluation of CD169 expression by flow cytometry (*n* = 3). A representative graph (upper) and geometric mean fluorescence intensity (GMFI) of CD169 (lower). **E**) Seventy-two hours after subcutaneous administration, inguinal LNs were harvested, and CD169 expression in CD11b^+^ F4/80^+^ cells was evaluated by flow cytometry (*n* = 4–6). The titer of mIFNα in each administration group was set to be equal by performing an in vitro experiment using J774.1 cells. A representative gating strategy for CD11b^+^ F4/80^+^ CD169^+^ cells (upper), CD169-positive macrophages (%) in CD11b^+^ F4/80^+^ cells (bottom left) and GMFI of CD169 in CD11b^+^ F4/80^+^ cells (bottom right). These results (B-E) are representative of two independent experiments. Data are averages ± S.E. *p < 0.05, **p < 0.01, ***p < 0.001, ****p < 0.0001, n.s.=nonsignificant; BV510 = Brilliant Violet 510; FVD780 = Fixable Viability Dye eFluor 780; PE = phycoerythrin; SSC-A = side scatter-peak area

To evaluate the lymphatic drainage of Man-MSA-mIFNα, we performed a similar experiment as in Fig. S10. Similar to MSA alone, over 20% of the ID per tissue (g) of both Man-MSA-mIFNα and MSA-mIFNα were distributed in the LN (Fig. 3A). Interestingly, a notable decline in radioactivity in both the MSA-mIFNα- and Man-MSA-mIFNα-administration groups was observed 6 h after the administration, indicating that they were taken up by the type I IFN receptor- or mannose receptor-expressing cells in the LNs [29, 34]. We additionally measured the radioactivity of Man-MSA-mIFNα in the main organs (heart, liver, kidney, lung, and spleen) and the blood, and found that Man-MSA-mIFNα showed remarkably low percentages of the ID per tissue (g) compared to that observed in the inguinal LN, indicating poor distribution in the main organs (Fig. S11). To further investigate whether Man-MSA-mIFNα has the capability to target macrophages, we subcutaneously administered Cy5-labeled Man-MSA-mIFNα to mice. An hour after the administration, fluorescence associated with Cy5-labeled Man-MSA-mIFNα and MSA-mIFNα was detected in the LN sinuses (Fig. 3B). Furthermore, Cy5-labeled Man-MSA-mIFNα was better incorporated into CD11b^+^ F4/80^+^ macrophages than Cy5-labeled MSA-mIFNα (Fig. 3C), whereas such an incorporation was hardly observed in LN dendric cells (Fig. S12). In contrast, the co-localization of Cy5-labeled Man-MSA-mIFNα with CD31^+^ lymphatic endothelial cells, which are components of the sinus, was significantly lower than that of Cy5-labeled MSA-mIFNα (Fig. S13). These results indicate that the target cell of Man-MSA-mIFNα is the macrophage.

To evaluate the ability of Man-MSA-mIFNα to induce the CD169^+^ phenotype, the expression of CD169 was analyzed by flow cytometry 24 h after the treatment of a mouse macrophage cell line, i.e., J774.1, with Man-MSA-mIFNα, MSA-mIFNα, or mIFNα. As we expected, Man-MSA-mIFNα, MSA-mIFNα, and mIFNα significantly induced CD169 expression in the cells (Fig. 3D). We then administered mIFNα, MSA-mIFNα, and Man-MSA-mIFNα subcutaneously to mice under the conditions where the titers of these proteins were set to be equal for evaluating the effects of Man-MSA-mIFNα on CD169^+^ phenotype in LN macrophages (Fig. S6). Even though the percentage of CD169^+^ macrophages was not changed among mIFNα, MSA-mIFNα, and Man-MSA-mIFNα treatment groups (Fig. 3E), Man-MSA-mIFNα resulted in significantly higher CD169 expression than both mIFNα and MSA-mIFNα after 72 h post administration (Fig. 3E). These data demonstrate that Man-MSA-mIFNα efficiently induced the strong expressed CD169^+^ phenotype in LN macrophages due to its high lymphatic drainage and macrophage-targeting ability.

## Antitumor effect of Man-MSA-mIFNα in tumor-bearing mice

**Fig. 4:**
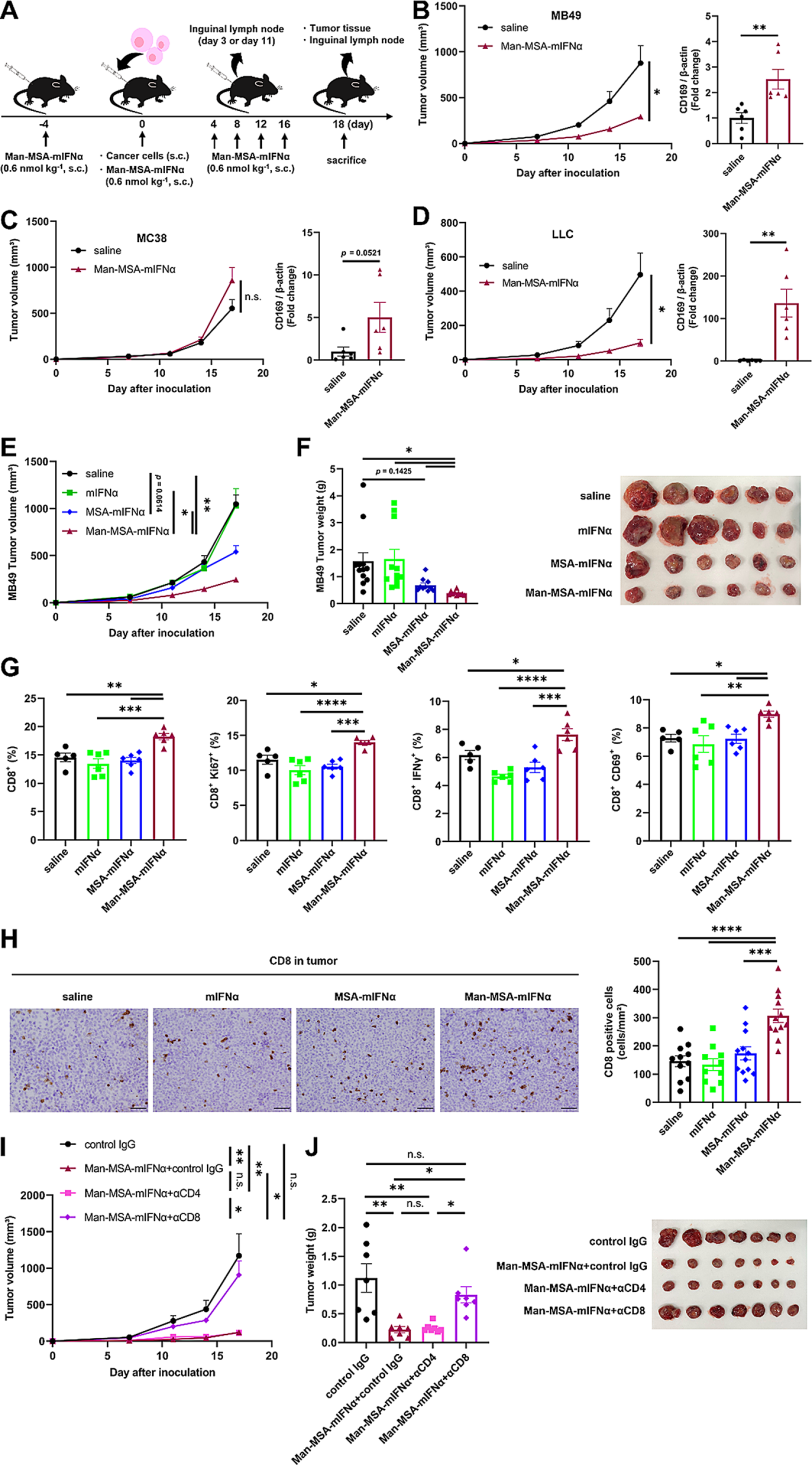
The effect of Man-MSA-mIFNα on tumor growth and CD8^+^ T cell activation in tumor-bearing mice. **A**) The experimental protocol for the evaluation of the antitumor effect of Man-MSA-mIFNα in tumor-bearing mice. **B**) Subcutaneous tumor volume was measured in MB49-bearing mice at each time point (*n* = 5–6). Inguinal LNs were harvested from MB49-bearing mice on the 3rd day after tumor inoculation and analyzed for CD169 expression by western blotting (*n* = 6). **C**) Subcutaneous tumor volume was measured in MC38-bearing mice at each time point (*n* = 6). Inguinal LNs were harvested from MC38-bearing mice on the 3rd day after tumor inoculation and analyzed for CD169 expression by western blotting (*n* = 6). **D**) Subcutaneous tumor volume was measured in LLC-bearing mice at each time point (*n* = 6). Inguinal LNs were harvested from LLC-bearing mice on the 11th day after tumor inoculation and analyzed for CD169 expression by western blotting (*n* = 6). These results (**B-D**) are representative of two independent experiments. **E**) Subcutaneous tumor volume was measured in MB49-bearing mice at each time point (*n* = 10–12). **F**) Subcutaneous tumor weight (*n* = 10–12) (left) and the representative image of tumors (right) on the 18th day after tumor inoculation. These results (**E, F**) represent combined data of two independent experiments. **G**) Inguinal LNs were harvested from MB49-bearing mice on the 18th day after tumor inoculation, followed by the evaluation of the percentage of CD8^+^ cells, Ki67^+^ CD8^+^ cells, IFNγ^+^ CD8^+^ cells, and CD69^+^ CD8^+^ cells in live cells by flow cytometry (*n* = 5–6). These results are representative of two independent experiments. **H**) Subcutaneous tumors on the 18th day after tumor inoculation were evaluated for tumor infiltration of CD8^+^ cells by immunohistochemistry (*n* = 10–12). Representative images (left) and quantification (right) of CD8^+^ cells in tumors. This result represents combined data of two independent experiments. Scale bar: 50 μm. **I**) Subcutaneous tumor volume was measured in MB49-bearing mice at each time point (*n* = 7). **J**) Subcutaneous tumor weight (*n* = 7) (left) and the representative image of tumors (right) on the 18th day after tumor inoculation. (**I, J**) MB49-bearing mice were depleted of CD4^+^ or CD8^+^ T cells and then received Man-MSA-mIFNα treatment. These results (**I, J**) are representative of two independent experiments. Data are averages ± S.E. *p < 0.05, **p < 0.01, ***p < 0.001, ****p < 0.0001, n.s.=nonsignificant

It is well known that immunogenic tumors are characterized by high T cell infiltration. In contrast, such infiltration is not observed in the case of non-immunogenic tumors [35]. To identify the types of cancers that Man-MSA-mIFNα could potentially be useful against, we examined the antitumor effects of Man-MSA-mIFNα on mice inoculated with a mouse bladder carcinoma cell line (MB49; a non-immunogenic tumor) or a mouse colon adenocarcinoma cell line (MC38; an immunogenic tumor) (Fig. 4A) [36, 37]. Man-MSA-mIFNα exerted significant antitumor effects against MB49-bearing mice, accompanied by an induction of CD169 expression in the LNs (Fig. 4B, Fig. S14A); however, no such effect was observed in MC38-bearing mice (Fig. 4C, Fig. S14B). As well as the case of MB49-bearing mice, the antitumor effects of Man-MSA-mIFNα were observed in mice bearing the murine Lewis lung carcinoma cell line (LLC; a non-immunogenic tumor) (Fig. 4D, Fig. S14C) [38]. Furthermore, some activation markers of CD8⁺ T cells were significantly increased in both MB49 and LLC-bearing mouse models in which Man-MSA-mIFNα treatment significantly inhibited tumor progression (Fig. S15A, C), whereas the activation markers of CD8⁺ T cells were unchanged in MC38-bearing mouse models in which Man-MSA-mIFNα treatment had no effect on tumor progression (Fig. S15B). Next, we compared the antitumor effects of Man-MSA-mIFNα with other mIFNα formulations using MB49-bearing mice. Consistent with the CD169 expression level shown in Fig. 3E, Man-MSA-mIFNα significantly inhibited tumor growth compared to mIFNα and MSA-mIFNα on the 18th day after tumor inoculation (Fig. 4E, F). Since the expression levels of CD169 contribute to the activation of CD8^+^ T cells, we further examined CD8^+^ T cell activation in LNs and the number of CD8^+^ T cells in tumor tissue. As expected, Man-MSA-mIFNα significantly increased the number of CD8^+^ T cells and boosted the expression levels of Ki67 and the production of IFNγ and CD69, both markers indicative of CD8^+^ T cell activation, in LNs (Fig. 4G). Consistent with the CD8^+^ T cell activation in LNs, Man-MSA-mIFNα substantially enhanced the infiltration of CD8^+^ T cells into the tumor (Fig. 4H). We further examined the inhibitory effect of Man-MSA-mIFNα on tumor progression in tumor-bearing mice model under CD8^+^ T cells depletion condition induced by anti-CD8 antibody treatment. As a result, anti-CD8 antibody treatment counteracted antitumor effect of Man-MSA-mIFNα in tumor-bearing mice model (Fig. 4I, J), whereas the antitumor effect of Man-MSA-mIFNα had little interference from anti-CD4 antibody treatment (Fig. 4I, J), indicating that CD8^+^ T cells induced by Man-MSA-mIFNα treatment is contributed to antitumor effect of Man-MSA-mIFNα.

## Antitumor effect of Man-MSA-mIFNα in CD169^+^ macrophage-depleted mice

**Fig. 5:**
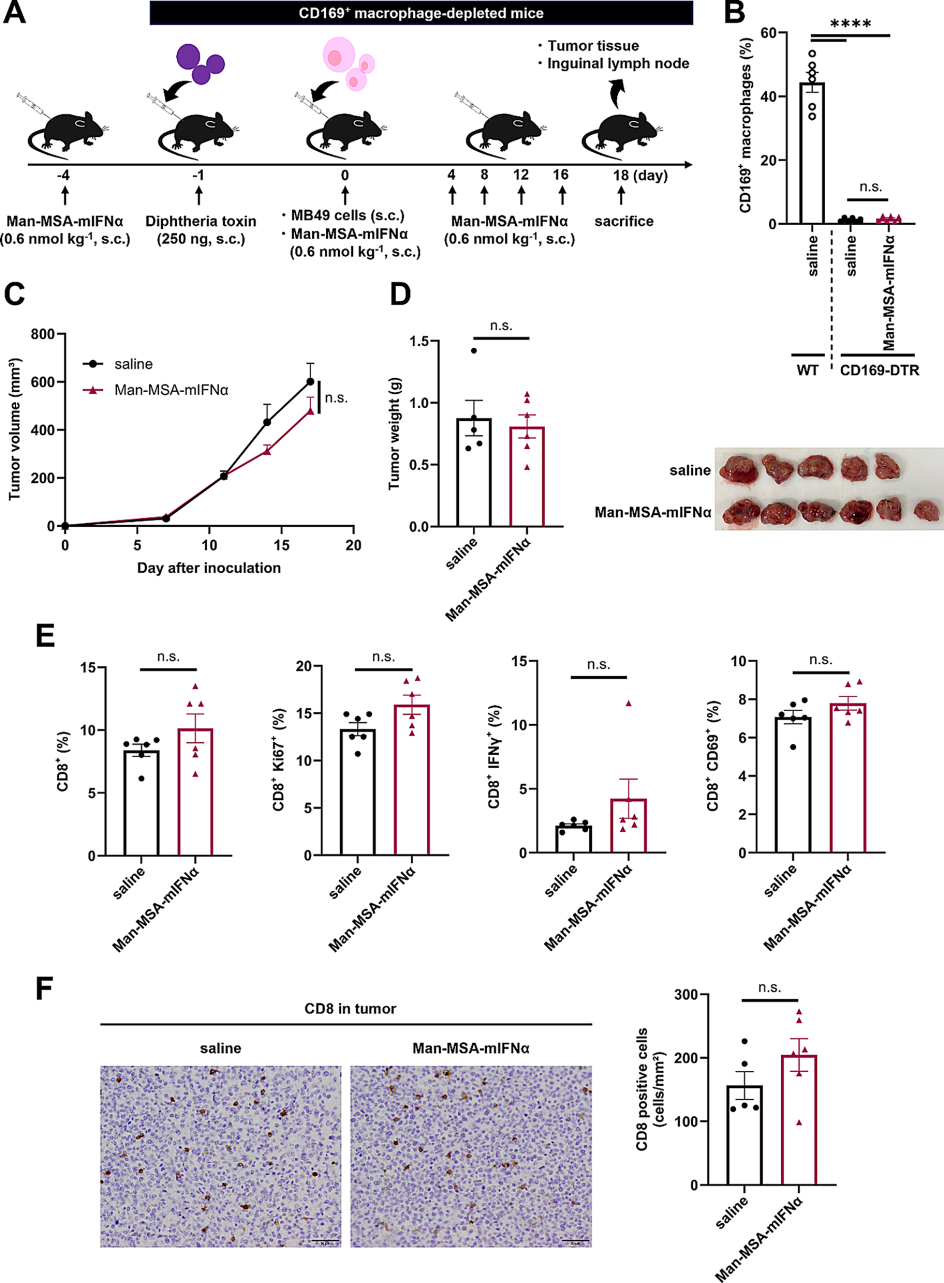
The effect of Man-MSA-mIFNα on tumor growth and CD8^+^ T cell activation in CD169^+^^**+**^ macrophage-depleted mice. **A**) The experimental protocol for the evaluation of the antitumor effect of Man-MSA-mIFNα in CD169^+^ macrophage-depleted MB49-bearing mice. **B**) Inguinal LNs were harvested from MB49-bearing WT mice or MB49-bearing CD169-DTR mice on the 18th day after tumor inoculation, followed by the evaluation of the percentage of CD169^+^ cells in CD11b^+^ F4/80^+^ cells by flow cytometry (*n* = 6). **C**) Subcutaneous tumor volume was measured in MB49-bearing mice at each time point (*n* = 5–6). **D**) Subcutaneous tumor weight (*n* = 5–6) (left) and the representative image of tumors (right) on the 18th day after tumor inoculation. **E**) Inguinal LNs were harvested from MB49-bearing mice on the 18th day after tumor inoculation, followed by the evaluation of the percentage of CD8^+^ cells, Ki67^+^ CD8^+^ cells, IFNγ^+^ CD8^+^ cells, and CD69^+^ CD8^+^ cells in live cells by flow cytometry (*n* = 6). **F**) Subcutaneous tumors on the 18th day after tumor inoculation were evaluated for tumor infiltration of CD8^+^ cells by immunohistochemistry (*n* = 5–6). Representative images (left) and quantification (right) of CD8^+^ cells in tumors. Scale bar: 50 μm. These results are representative of two independent experiments. Data are averages ± S.E. ****p < 0.0001, n.s.= nonsignificant

To demonstrate whether the antitumor effect of Man-MSA-mIFNα is dependent on LN CD169^+^ macrophages, we first examined the inhibitory effect of Man-MSA-mIFNα on tumor progression in tumor-bearing mice model under the condition of removing tumor-draining LN (Fig. S16A). As a result, removing the tumor-draining LN counteracted the antitumor effect of Man-MSA-mIFNα in tumor-bearing mice model (Fig. S16B, C), indicating that tumor-draining LN plays a critical role in the antitumor effect of Man-MSA-mIFNα. We next used CD169-DTR mice in which LN CD169^+^ macrophages can be depleted by the subcutaneous injection of diphtheria toxin (DT) (Fig. 5A) [4]. Under the conditions where the number of CD169^+^ macrophages in the LNs was significantly lowered by DT injection (Fig. 5B), the administration of Man-MSA-mIFNα did not decrease the tumor volume and weight of MB49-bearing CD169-DTR mice compared to those of the saline-treated group (Fig. 5C, D). In addition, Man-MSA-mIFNα treatment also did not affect CD8^+^ T cell activation and infiltration in tumor tissues in CD169-DTR mice (Fig. 5E, F). These findings show that the antitumor effects of Man-MSA-mIFNα were mediated by its activation of CD8^+^ T cells via the induction of the CD169^+^ phenotype of LN macrophages.

### Antitumor effect of a combination therapy of Man-MSA-mIFNα and PD-L1 Blockade

**Fig. 6:**
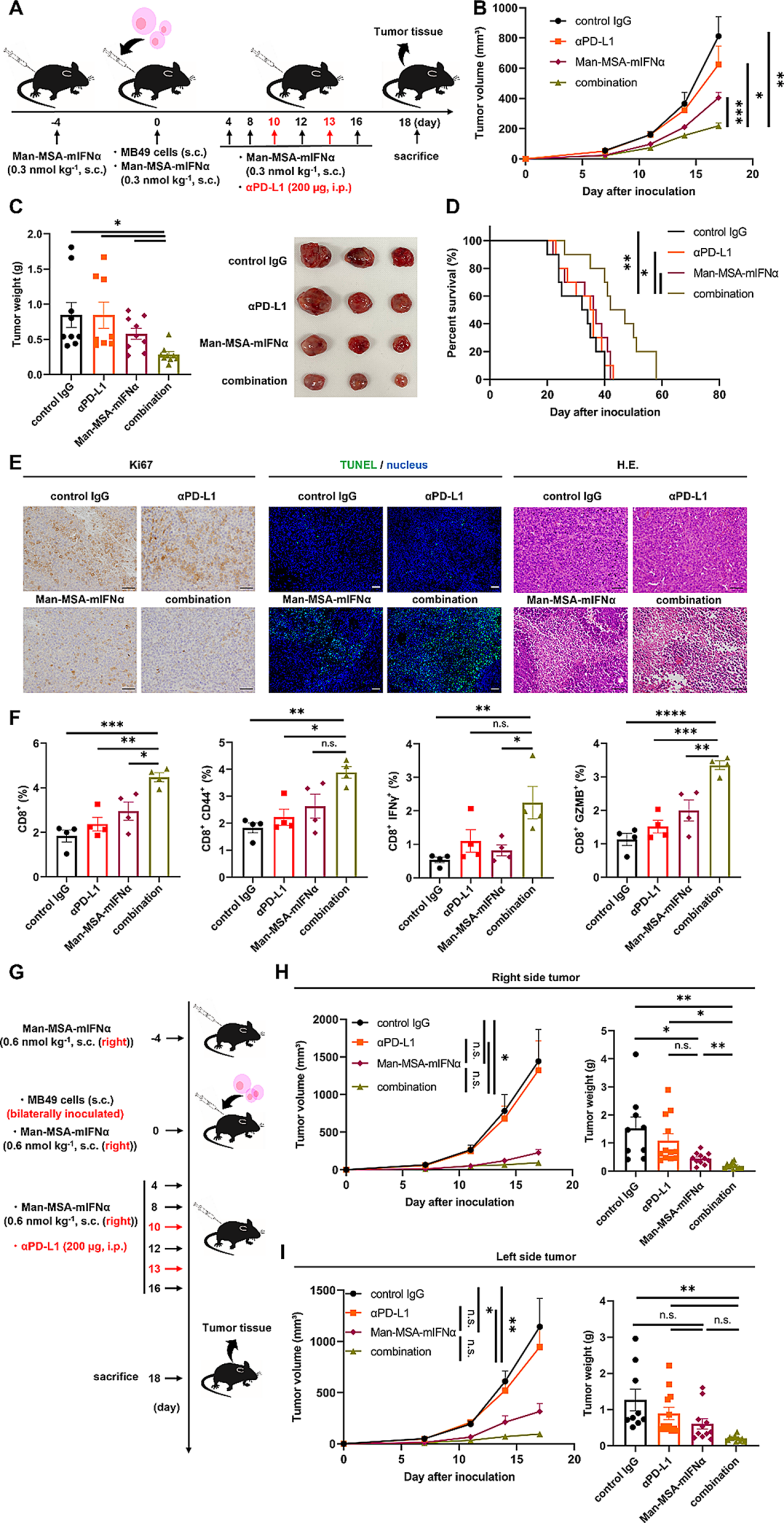
**The effect of combination therapy on tumor growth and CD8**^**+**^**T cell activation in tumor-bearing mice. A**) The experimental protocol for the evaluation of the antitumor effect of combination therapy in MB49-bearing mice. **B**) Subcutaneous tumor volume was measured in MB49-bearing mice at each time point (*n* = 8–9). **C**) Subcutaneous tumor weight (*n* = 8–9) (left) and the representative image of tumors (right) on the 18th day after tumor inoculation. These results (B, C) represent combined data of two independent experiments. **D**) Survival time was observed in MB49-bearing mice treated by PD-L1 blockade and/or Man-MSA-mIFNα (*n* = 10). This result is representative of two independent experiments. **E**) Representative images of immunostaining for Ki67, terminal deoxynucleotidyl transferase dUTP nick end labeling (TUNEL), and H.E. staining of tumors from MB49-bearing mice on the 18th day after tumor inoculation. Scale bar: 50 μm. **F**) Subcutaneous tumors were harvested from MB49-bearing mice on the 18th day after tumor inoculation, followed by the evaluation of the percentage of CD8^+^ cells, CD44^+^ CD8^+^ cells, IFNγ^+^ CD8^+^ cells, and GZMB^+^ CD8^+^ cells in live cells by flow cytometry (*n* = 4). These results (E, F) are representative of two independent experiments. **G**) The experimental protocol for the evaluation of the antitumor effect in bilateral MB49-bearing mice. Subcutaneous tumor volume and weight were measured in MB49-bearing mice at both the **H**) local tumor (right side tumor) and the **I**) distant tumor (left side tumor) (*n* = 9–12). These results (H, I) represent combined data of two independent experiments. Data are averages ± S.E. *p < 0.05, **p < 0.01, ***p < 0.001, ****p < 0.0001, n.s.= nonsignificant; αPD-L1 = alpha programmed death-ligand 1

Since the remarkable antitumor effects of immune checkpoint inhibitors are attributed to the suppression of CD8^+^ T cell exhaustion signaling, these treatments may not be as beneficial for cancer patients exhibiting minimal CD8^+^ T cell infiltration into tumors [39]. However, Man-MSA-mIFNα has demonstrated an ability to boost antitumor immunity and increase CD8^+^ T cell infiltration into tumor tissues substantially. Consequently, we speculated that the combination of Man-MSA-mIFNα and immune checkpoint inhibitors could ameliorate treatment resistance in patients who are unresponsive to immune checkpoint inhibitors. To confirm this hypothesis, we evaluated the antitumor effects of a combination therapy using MB49-bearing mice, which are known to exhibit resistance to PD-L1 blockade monotherapy (Fig. 6A) [40]. Here, the dose of Man-MSA-mIFNα was set at half (0.3 nmol kg^− 1^) compared to the previous experiment (0.6 nmol kg^− 1^), because it was expected that the antitumor effect of Man-MSA-mIFNα would be enhanced by combination therapy with PD-L1 blockade. As expected, no significant treatment effect was observed with the PD-L1 blockade alone (Fig. 6B, C). In contrast, the combination therapy showed a significant antitumor effect compared to the PD-L1 blockade or Man-MSA-mIFNα alone. Furthermore, the combination therapy of Man-MSA-mIFNα with anti-PD-L1 antibody significantly extended overall survival compared to the PD-L1 blockade or Man-MSA-mIFNα alone (Fig. 6D). Consistent with these results, we observed a decrease in Ki67^+^ cells, a marker for tumor growth, and an increase in apoptotic cells in the tumor tissues after the combination therapy (Fig. 6E). Furthermore, the number of activated CD8^+^ T cell in the tumor tissue showed a significant increase following combination therapy (Fig. 6F). We further examined the systemic antitumor effect of combination therapy of Man-MSA-mIFNα with anti-PD-L1 antibody in bilateral tumor-bearing mice model. Man-MSA-mIFNα or combination of Man-MSA-mIFNα and PD-L1 antibody were injected subcutaneously near the right tumor (local tumor) once every 4 days while monitoring the sizes of the left tumor (distant tumor) (Fig. 6G). Man-MSA-mIFNα alone and the combination therapy inhibited tumor progression in both the local and distant tumors (Fig. 6H, I), suggesting that Man-MSA-mIFNα-based cancer immunotherapy targeting CD169⁺ macrophages in regional LN also leads to distant tumor suppression. Taken together, these results suggest that Man-MSA-mIFNα functioned as an “immune booster” that attenuated therapeutic resistance to immune checkpoint inhibitors by increasing CD8^+^ T cell infiltration into tumor tissues.

### Safety evaluation

Given that the administration of IFNα induces a variety of side effects such as gastrointestinal symptoms, leukopenia, thrombocytopenia, anemia, liver damage, and renal damage [41], there are concerns that Man-MSA-mIFNα administration might induce similar adverse reactions. To investigate this possibility, we assessed several IFNα-associated side effects in tumor-bearing mice that had been treated with Man-MSA-mIFNα. As shown in Fig. S17A, no body weight loss or reduction in white blood cells (WBCs) and platelets (PLTs) was observed in any treatment group. In addition, similar results were obtained for red blood cells (RBCs), hemoglobin (HGB), and the hematocrit value (HCT). The levels of serum alanine aminotransferase (ALT), aspartate aminotransferase (AST), and blood urea nitrogen (BUN) showed no abnormal values in any group. H.E. staining also revealed no tissue damage (Fig. S17B). These findings suggest that, under our experimental conditions where significant antitumor effects were observed in the Man-MSA-mIFNα-treated group (7-week-old male C57BL/6 N mice), Man-MSA-mIFNα is unlikely to elicit side effects related to IFNα, such as weight loss, bone marrow suppression, or organ damage.

## Discussion

Our findings revealed significant compositional patterns in regional LNs, specifically that they were rich in macrophages, as well as CD8^+^ and CD4^+^ T cells. Notably, the Pearson correlation coefficients calculated for cell numbers within neighborhoods showed a positive correlation between the number of macrophages and the number of CD8^+^ (*r* = 0.57) and CD4^+^ T cells (*r* = 0.76). This data suggests the existence of specific regions where CD169^+^ macrophages may play a crucial role in priming T cells within the regional LNs of cancer patients. These discoveries are important as they may deepen our understanding of immune interactions in the LN microenvironment and could have implications for cancer immunotherapy strategies. Further research is necessary to elucidate the underlying mechanisms of these interactions and to explore potential clinical applications that could enhance therapeutic outcomes for patients.

Lymphatic drainage of subcutaneously administered substances is regulated by molecular size (5–100 nm), molecular weight (> 20 kDa), and zeta potential (negatively charged surfaces) [42]. Interestingly, albumin, which is widely used as a drug delivery system (DDS) carrier for prolonging the circulation time of drugs and proteins, is also qualified as a LN-targeted DDS carrier due to its fascinating molecular properties—10 nm (diameter), 66.5 kDa (molecular mass), and negatively charged surface. Our pharmacokinetic experiments using radioactive iodine demonstrated that albumin-fused mIFNα gained ~ 1.6 times greater access to a LN than mIFNα alone (Fig. S10 and Fig. 3A). Interstitial oncotic pressure also contributes to lymphatic drainage. Trubetskoy et al. found that massaging the injection site of liposomes enhanced the lymphatic absorption of liposomes [43]. Albumin also increases the interstitial pressure at the injection site due to its osmoregulatory function. In fact, subcutaneously administered HSA mixed with IFNα improves the lymphatic absorption of the IFNα [44]. Based on these unique properties, it is reasonable to infer that albumin is a suitable DDS carrier to target LNs.

In the present study, we successfully applied Man-MSA as a DDS to target LN macrophages and induced the CD169^+^ phenotype of macrophages by delivering IFNα to the cells (Fig. 3). Resident conventional dendritic cells (cDCs) in LNs also contribute to CTL activation via antigen presentation [45]. The IFNα response further enhances their functions through the increased expression of CD80/86 (co-stimulatory molecules) and major histocompatibility complex class I. Given that DCs, like macrophages, express mannose and type I IFN receptors, it is possible that the antitumor effect of Man-MSA-mIFNα resulted from an off-target effect on cDCs. However, it is unlikely that Man-MSA-mIFNα would distribute to cDCs because of the structural features of LNs. LNs are structurally classified into the cortex, paracortex, and medulla. CD169^+^ macrophages are located in the subcapsular (cortex) and medullary (medulla) sinuses adjacent to afferent and efferent lymphatics [46]. In contrast, cDCs are located in the paracortex, deep in the LNs [47]. To gain access deep in the LN, lymph-borne molecules must flow through the conduit, branching out from the lymphatic sinuses. Since the conduit is very narrow (3–5 nm), only molecules smaller than 70 kDa can reach deep inside the LN [48]. Our results showed that Cy5-labeled Man-MSA-mIFNα (88.5 kDa) localized in the subcapsular and medullary sinuses of a LN with few cDCs and abundant CD169^+^ macrophages (Fig. 3B). These data corroborate the fact that not only is Man-MSA highly mannosylated but it also has the desired molecular size to target LN macrophages.

Asano et al. subcutaneously administered dead cancer cells to mice and found that they were taken up by CD169^+^ macrophages in the LNs [4]. Furthermore, as mentioned above, the fusion of Man-MSA and mIFNα improved the distribution of mIFNα to LNs and CD169^+^ macrophages and efficiently induced CD169 expression (Fig. 3). These results show that LN CD169^+^ macrophages internalized cancer antigens that had been distributed to the LNs, and the subsequent process of antigen presentation to T cells was likely boosted by Man-MSA-mIFNα. Here, to achieve the antitumor effect of Man-MSA-mIFNα, it must be injected subcutaneously near the tumor and delivered to LN macrophages exposed to the cancer antigens. However, given the wide variety of carcinomas, it is challenging to administer drugs subcutaneously in the vicinity of the tumor in an actual clinical setting. Therefore, in the future, it may be necessary to broaden the subcutaneous administration sites from ‘near the tumor’ to ‘throughout the body’ by incorporating not only mIFNα but also cancer antigens into Man-MSA.

In this study, we prepared tumor-bearing mice with immunogenic tumors (MC38) or non-immunogenic tumors (MB49 and LLC), each exhibiting different levels of tumor immunogenicity. Interestingly, Man-MSA-mIFNα treatment exerted a significant antitumor effect and remarkably enhanced the CD169^+^ phenotype in LN macrophages only in non-immunogenic tumor-bearing mice (Fig. 4B-D). CD169 expression has been used as a surrogate biomarker for type I interferon activity in many diseases [49]. Therefore, we speculate that the difference in the antitumor effect of Man-MSA-mIFNα between immunogenic and non-immunogenic tumors was due to the varying endogenous IFNα expression levels in these two types of cancer. In other words, the CD169 expression levels of LN macrophages would be highly regulated in immunogenic tumors with high endogenous IFNα levels. As a result, Man-MSA-mIFNα failed to induce the CD169^+^ phenotype of macrophages in LNs, not yielding any antitumor effect. In the clinical setting, the numbers of CD169^+^ macrophages in LNs are known to be different in patients even with the same cancer type [26]. Therefore, our IFNα formulation could potentially be beneficial for cancer patients pathologically diagnosed with low CD169 expression levels via LN biopsy. In the future, it will be necessary to develop non-invasive methods for monitoring LN CD169, such as liquid biopsy for IFNα, to tailor individual cancer immunotherapy based on the CD169^+^ macrophage content.

The present study successfully achieved our therapeutic strategy of “the induction of the CD169^+^ phenotype in LN macrophages” by active targeting of IFNα to LN macrophages. Therefore, it can be expected that this cancer immunotherapy allows for the combination of Man-MSA-mIFNα with not only immune checkpoint inhibitors but also other immune-enhancing drugs, such as CAR-T therapy, radiotherapy, and anticancer drugs, for the treatment of intractable cancers that are challenging to treat with single agents or existing combination therapies. Further, we previously reported the development of a genetic fusion of human IFNα and mannosylated human serum albumin (Man-HSA-hIFNα) [50]. The present study demonstrated that Man-HSA-hIFNα induces the CD169^+^ phenotype in human monocyte-derived macrophages (Fig. S18). Therefore, Man-HSA-hIFNα, optimized through our drug discovery technologies, is expected to exert a significant antitumor effect in humans.

## Conclusions

The present study successfully designed and developed Man-MSA-mIFNα, which efficiently induces the CD169^+^ phenotype in LN macrophages, contributing to the antitumor immune response. The findings of this study not only reveal the usefulness of a therapeutic strategy targeting CD169⁺ macrophages in the LNs but also suggest that Man-HSA-hIFNα could be a promising immune booster for cancer patients who are unresponsive to immune checkpoint inhibitors.

## Electronic supplementary material

Below is the link to the electronic supplementary material.

Supplementary Material 1

Supplementary Material 2

## Data Availability

The datasets used and/or analyzed during the current study are available from the corresponding author on reasonable request.

## Abbreviations

AGP: α1-acid glycoprotein
ALT: Alanine aminotransferase
AST: Aspartate aminotransferase
BUN: Blood urea nitrogen
CBB: Coomassie Brilliant Blue
CD: Circular dichroism
cDC: Conventional dendritic cell
CTL: Cytotoxic T lymphocyte
DDS: Drug delivery system
DT: Diphtheria toxin
GMFI: Geometric mean fluorescence intensity
HCT: Hematocrit
H.E.: Hematoxylin-eosin
HGB: Hemoglobin
ID: Initial dose
IFN: Interferon
IFNAR1: Interferon alpha/beta receptor 1
LLC: Lewis lung carcinoma
LN: Lymph node
Man-HSA: Mannosylated human serum albumin
Man-MSA: Mannosylated mouse serum albumin
mIFNα: Mouse interferon alpha
PAS: Periodic acid-Schiff
PD-1: Programmed death-1
PD-L1: Programmed death-ligand 1
PLT: Platelet
RBC: Red blood cell
s.c.: Subcutaneous
SDS-PAGE: Sodium dodecyl sulfate-polyacrylamide gel electrophoresis
TUNEL: Terminal deoxynucleotidyl transferase dUTP nick end labeling
WBC: White blood cell
^125^I: Iodine-125
