# Supplementary material for: Lymph node macrophage-targeted interferon alpha boosts anticancer immune responses by regulating CD169-positive phenotype of macrophages
Source: Mol Cancer. 2025 May 3;24:132. doi: 10.1186/s12943-025-02324-8 (PMC12049019; doi:10.1186/s12943-025-02324-8)
Supplement: Supplementary file 1 — Supplementary Material 1 [file 12943_2025_2324_MOESM1_ESM.docx]

**Supplementary Contents**

**Lymph node macrophage-targeted interferon alpha boosts anticancer immune responses by regulating CD169-positive phenotype of macrophages**

**Table of contents**

**Supplementary Methods**

- Materials
- LN samples
- Cells and animals
- Immunofluorescence staining
- Immunohistochemistry
- Expression and purification of fusion proteins
- SDS-PAGE and western blotting
- CD spectra
- Biosafety evaluation

**Supplementary Figures**

- Fig. S1. Flow chart describing the construction of MSA (D494N)-mIFNα2 (N78Q) gene using pPIC9.
- Fig. S2. A representative gating strategy for CD169^+^ macrophages and CTLs in LN.
- Fig. S3. A representative gating strategy for CTLs in tumor.
- Fig. S4. The optimal dose of Man-MSA-mIFNα for CD169 induction in vivo.
- Fig. S5. The time course of CD169 expression in vivo.
- Fig. S6. The titer of mIFNα or Man-MSA-mIFNα for CD169 induction in vitro.
- Fig. S7. Evaluation of the optimal dosing interval using MB49-bearing mice.
- Fig. S8. The cell composition of neighborhoods in CytoMAP and the P value of Pearson correlation coefficients.
- Fig. S9. Expression of mannose receptor and type Ⅰ IFN receptor in LN macrophages.
- Fig. S10. Lymphatic drainage of albumin.
- Fig. S11. Pharmacokinetic analysis of ^125^I-labeled Man-MSA-mIFNα.
- Fig. S12. Distribution of Man-MSA-mIFNα to LN dendritic cells.
- Fig. S13. IntraLN distribution of Man-MSA-mIFNα.
- Fig. S14. CD169 induction by Man-MSA-mIFNα in tumor-draining LN.
- Fig. S15. Effect of Man-MSA-mIFNα on the phenotype of CD8⁺ T cells in LN.
- Fig. S16. Effect of Man-MSA-mIFNα on tumor progression under the condition of removing the tumor-draining LN.
- Fig. S17. Effect of Man-MSA-mIFNα on digestive symptoms, hematopoietic system, serum parameters and organ in MB49-bearing mice.
- Fig. S18. CD169 induction by Man-HSA-hIFNα in human monocyte-derived macrophages.

**Supplementary Tables**

- Table S1. The list of antibodies for experiments.
- Table S2. Sequence of mutagenic primers for point mutations of MSA (D494N) or mIFNα2 (N78Q).

***Materials***: AGP was purified from the supernatant of human plasma fraction V provided by KM Biologics Co., Ltd. ^125^I was purchased from PerkinElmer (Waltham, MA). MSA was purchased from Biomedical Science (Tokyo, Japan). Recombinant mIFNα (carrier-free) for pharmacokinetic analysis was purchased from BioLegend (San Diego, CA). mIFNα was purchased from Wako (Osaka, Japan) for the evaluation of CD169 expression. hIFNα was purchased from Sigma-Aldrich (St Louis, MO).

***LN samples***: Samples of intestinal LNs were surgically collected from patients with colorectal cancer who were diagnosed at Izumi General Hospital (Izumi, Kagoshima, Japan). Nonmetastatic specimens were enrolled in this study. Tissue samples were fixed with 10% neutral buffered formalin and embedded in paraffin. Written informed consent was obtained from all patients, and the study design was approved by the review board (#57).

***Cells and animals***: Cell lines, including J774.1 (mouse macrophage) and MB49 (mouse bladder cancer) were purchased from RIKEN Cell Bank (Tsukuba, Japan) or JCRB Cell Bank (Tokyo, Japan). MC38, a mouse colon cancer cell line, was purchased from Kerafast (Boston, MA). A LLC cell line was kindly gifted by Keizo Takenaga (Chiba Cancer Center, Japan). J774.1 cells, MB49 cells, MC38 cells, and LLC cells were cultured in RPMI 1640 (Wako) containing 10% fetal bovine serum (FBS, Wako) and 1% penicillin/streptomycin (Wako) at 37 °C in a 5% CO₂ atmosphere.

Blood was collected after obtaining written informed consent from all donors of peripheral blood mononuclear cells (PBMCs) and the experimental procedure was approved by the Institutional Review Board of Kumamoto University (#1169). PBMCs were isolated using a lymphocyte separation solution (Lymphoprep™, Cosmo Bio, Tokyo, Japan) from the buffy coats of healthy donors. Monocytes were cultured in DMEM supplemented with 2% FBS, 1% penicillin/streptomycin, and granulocyte macrophage-colony-stimulating factor (5 ng mL^−1^) for 3 days for differentiation into human monocyte-derived macrophages.

Three-week-old male ICR mice were purchased from Japan SLC, Inc. (Shizuoka, Japan) and 4-week-old male C57BL/6N mice from CLEA Japan (Tokyo, Japan). CD169-DTR mice were kindly gifted by Kenichi Asano (Yokohama City University, Japan). C57BL/6N mice were subcutaneously inoculated in the inguinal region with MC38 (5 × 10^5^ cells/mouse), MB49 (2 × 10^5^ cells/mouse), or LLC (3 × 10^5^ cells/mouse) suspended in phosphate-buffered saline (PBS, 100 μL). To deplete inguinal LN-CD169^+^ macrophages in CD169-DTR mice, diphtheria toxin (Wako) was administered subcutaneously (250 ng) into the inguinal region the day before the tumor inoculation. The mice were housed in a temperature-controlled room with a 12 hour light-dark cycle and given free access to food and water. All animal experiments were approved by the experimental animal ethics committee at Kumamoto University (A2021-021) and conducted in accordance with the Institutional Animal Care and Use Committee.

***Immunofluorescence staining***: Inguinal LNs were harvested and frozen in Tissue-Tek OCT compound (Sakura Finetek; Torrance, CA). After cryosectioning, the 4 µm-thick frozen sections were fixed in 4% paraformaldehyde for 15 min, washed with TB (50 mM Tris-HCl, pH 7.5), blocked with 4% Block ACE (KAC Co., Ltd, Tokyo, Japan) at room temperature for 10 min, and incubated overnight with primary antibodies at 4 °C. After washing with TB, the sections were incubated with secondary antibodies at room temperature for 90 min. For the detection of apoptotic cells in tumor tissues, terminal deoxynucleotidyl transferase dUTP nick end labeling (TUNEL) was performed using an In Situ Cell Death Detection Kit and fluorescein (Roche, Basel, Switzerland). Fluorescence images were obtained using a fluorescence microscope (BZ-X700; Keyence). The percentage of Cy5^+^ cells in the total number of CD31^+^ cells per field of view was calculated using BZ-X analyzer software (Keyence) based on six random images taken at 200x magnification. The antibodies are described in Table S1.

***Immunohistochemistry***: Tissue samples were fixed in 10% neutral buffered formalin at room temperature for 24 hours and then embedded in paraffin wax. The paraffin blocks were sliced into 3 µm-thick sections, and the sections were deparaffinized in xylene and rehydrated in a graded ethanol series. After heating in a pressure cooker for antigen retrieval, the sections were incubated overnight with primary antibodies at 4 °C and then with horseradish peroxidase (HRP)-labeled secondary antibodies at room temperature for 30 min before being visualized using the 3,3’-diaminobenzidine (DAB) substrate system (NICHIREI BIOSCIENCES INC., Tokyo, Japan). For double staining of LN, the sections were stained with anti-CD169 and anti-CD68 antibodies as described above, and visualized with DAB and HistoGreen (Linaris, Frankfurt, Germany), respectively. The antibodies are described in Table S1. CD8^+^ cells per field of view were calculated using ImageJ based on six random images taken at 200x magnification.

***Expression and purification of fusion proteins***: Large-scale cultures of Pichia pastoris were prepared by shaking 5 L of BMGY medium (1% yeast extract, 2% peptone, potassium phosphate [100 mM, pH 6.0], 1.34% yeast nitrogen base with ammonium sulfate, 4 × 10^−5^% biotin, 1% glycerol; growing phase) for 2 days at 27 °C. Then, the medium was changed to 800 mL of BMMY medium (1% yeast extract, 2% peptone, potassium phosphate [100 mM, pH 6.0], 1.34% yeast nitrogen base with ammonium sulfate without amino acids, 4 × 10^−5^% biotin, 1% methanol; protein induction phase) and the cells were cultured for 3 days at 25 °C. Methanol was used as a carbon source to induce protein expression and added to a final concentration of 1% every 24 hours. The culture supernatant was dialyzed against deionized water, concentrated, replaced with acetate buffer (200 mM, pH 5.5), and then applied to a Blue Sepharose 6-Fast Flow column (Cytiva, Tokyo, Japan). KSCN (2 M) in acetate buffer (200 mM, pH 5.5) was used to elute the protein. After dialysis and concentration, the fraction eluted by KSCN was replaced with binding buffer (Na₃PO₄ (20 mM), NaCl (500 mM), and imidazole (20 mM), pH 7.4) and applied to a HisTrap HP column (Cytiva) connected to an AKTA prime device. The eluted fraction was collected using elution buffer (Na₃PO₄ (20 mM), NaCl (500 mM), and imidazole (500 mM), pH 7.4). After dialysis and concentration, the fusion proteins were lyophilized and stored at −20 °C.

***SDS-PAGE and western blotting***: For physicochemical analysis, SDS-PAGE was performed using a 10% polyacrylamide gel. The fusion proteins were visualized by staining with CBB R-25, while the glycoproteins were observed by PAS staining. The gel was fixed in 50% methanol for 30 min, washed with 3% acetic acid, incubated with 1% periodic acid for 15 min, and then stained with Schiff's reagent for 15 min. To perform western blotting after electrophoresis, the resolved proteins in the gel were transferred to a hydrophilized polyvinylidene fluoride (PVDF) membrane for 10 min at 1.3 A, 25 V using a trans-blot Turbo system (Bio-Rad, Hercules, CA). The PVDF membrane was blocked with 5% skim milk in Tris-buffered saline (TBS) containing 0.1% Tween 20 (TBS/T) at room temperature for 1 hour. After washing with TBS, the membrane was incubated with primary antibodies at 4 °C overnight and then, with secondary antibodies at room temperature for 1 hour after washing with TBS/T. SuperSignal Western blotting detection reagents (Thermo Scientific, Rockford, IL) were used to visualize the proteins. For the assessment of CD169 expression in LNs, the total protein was extracted using a mammalian protein extraction reagent (M-PER, Thermo Scientific) containing a 1% protease inhibitor cocktail (Nacalai Tesque, Kyoto, Japan), and the lysate was centrifuged at 6,700 g for 5 min at 4 °C. Protein concentrations in the supernatants were measured using a bicinchoninic protein assay kit (Pierce Chemical Co., Rockford, IL). The proteins were then incubated with 6x sample buffer (Tris-HCl [0.375 M], pH 6.8, 60% glycerol, 0.12% bromophenol blue, DTT [0.6 M], and 30% sodium dodecyl sulfate) for 30 min at 37 °C before being loaded onto a 7% polyacrylamide gel. The subsequent steps were performed in the same manner as described above. The data were quantified using ImageJ software. The antibodies are described in Table S1.

***CD spectra***: A Jasco J-820 spectropolarimeter (JASCO, Tokyo, Japan) was used to investigate the secondary or tertiary structure of the fusion protein. The proteins were dissolved in PBS (pH 7.4), their concentration was adjusted to 2 µM, and spectra acquired at 25 °C.

***Biosafety evaluation***: The main organs and blood were collected from MB49-bearing mice on the 18th day after tumor inoculation. The main organs were fixed by treatment with 10% neutral buffered formalin at room temperature for 24 hours to perform H.E. staining. Blood cell parameters were measured using a blood cell measuring instrument (SYSMEX Co., Hyogo, Japan). Plasma was obtained by centrifuging the blood at 2,000 g for 10 min, and then AST, ALT, and BUN levels were measured. All markers were measured using commercially available assay kits (Wako).

**
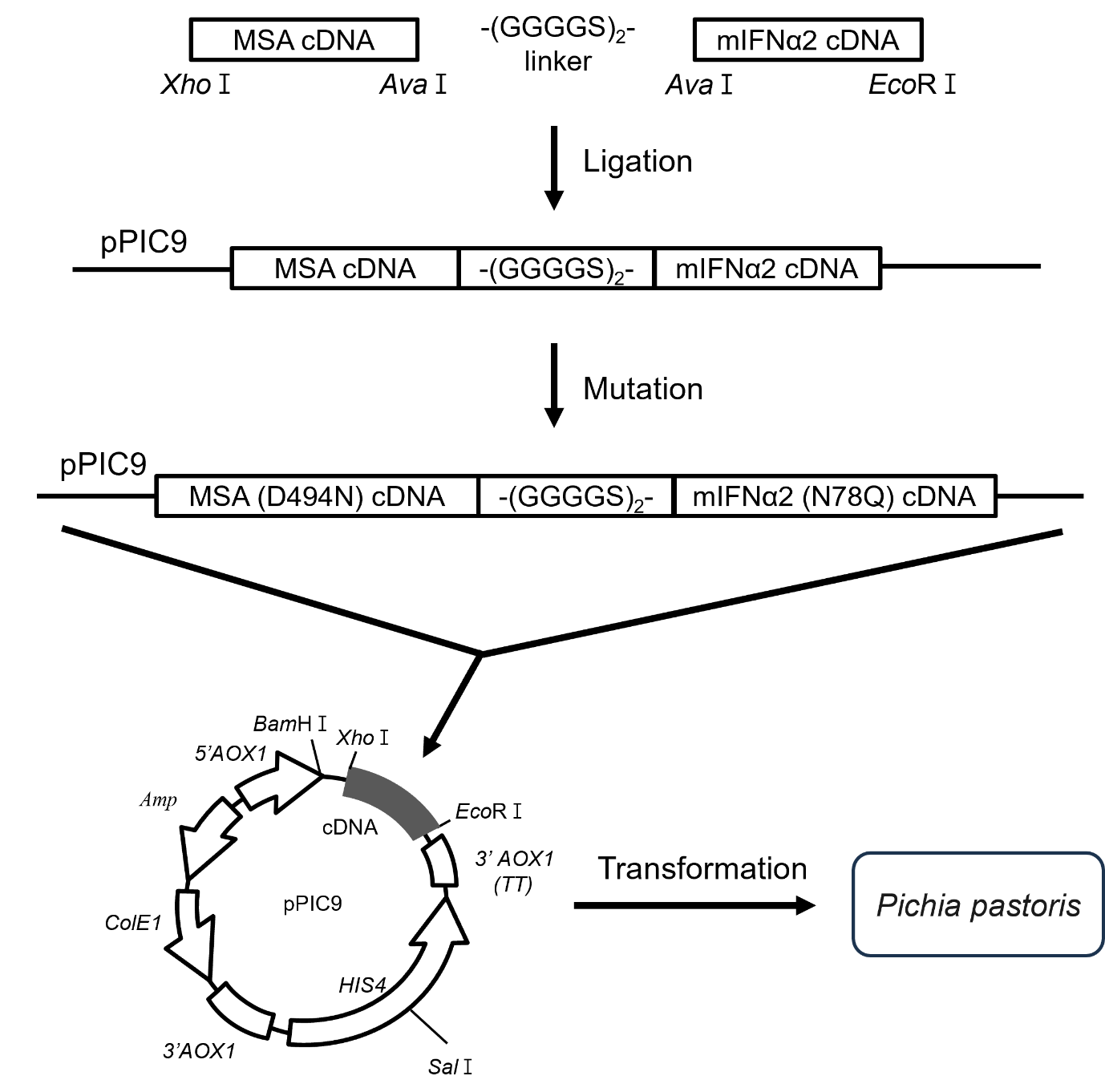
Supplementary Fig. S1**

**Fig. S1. Flow chart describing the construction of MSA (D494N)-mIFNα2 (N78Q) gene using pPIC9.**

**
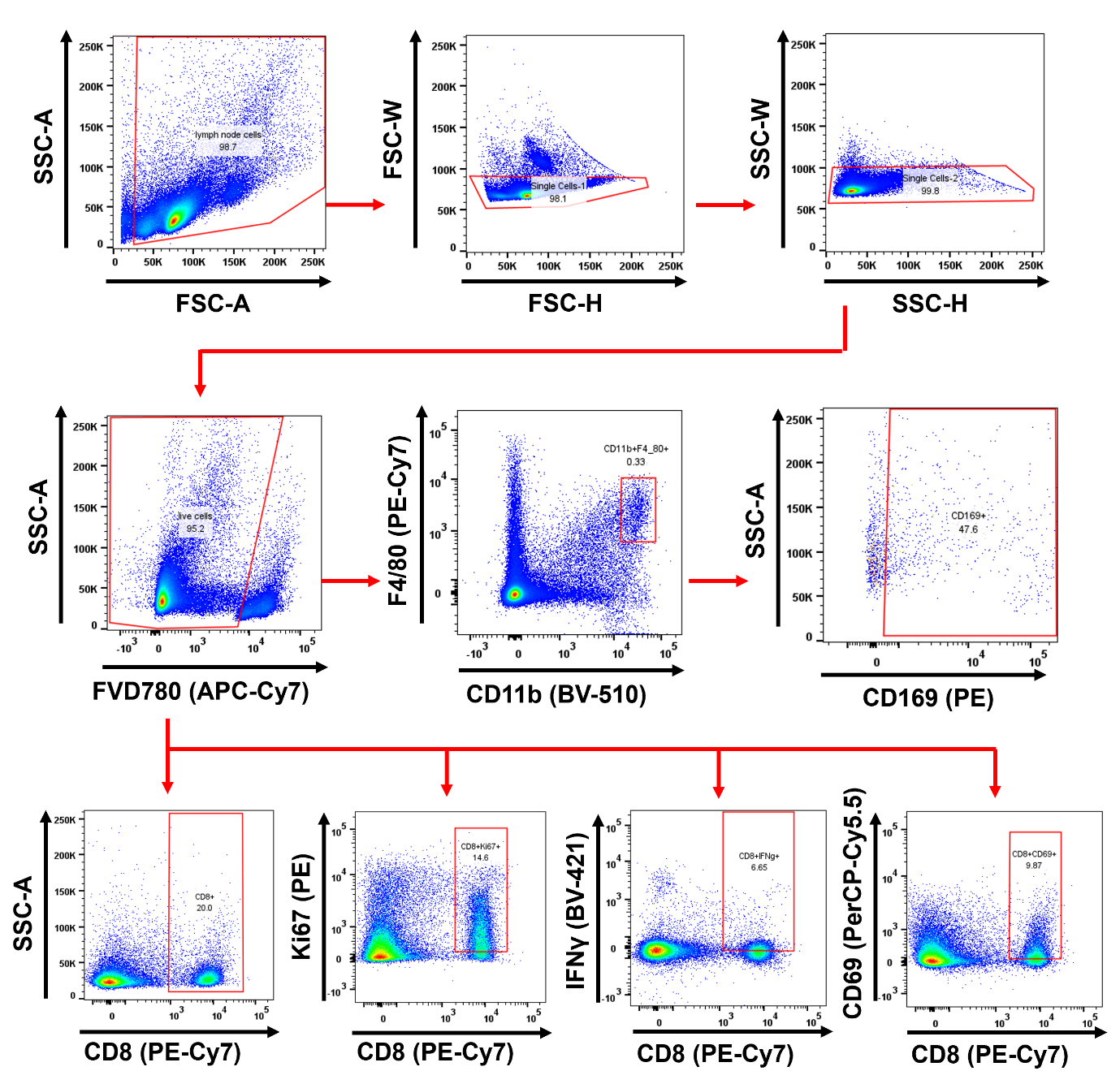
Supplementary Fig. S2**

**Fig. S2. A representative gating strategy for CD169^+^ macrophages and CTLs in LN.**

To deplete dead cells, single LN cells were stained using Fixable Viability Dye eFluor 780 (FVD780). The live cells were stained by antibodies against CD11b and F4/80 to identify macrophages and against CD8 to identify CTLs.

**
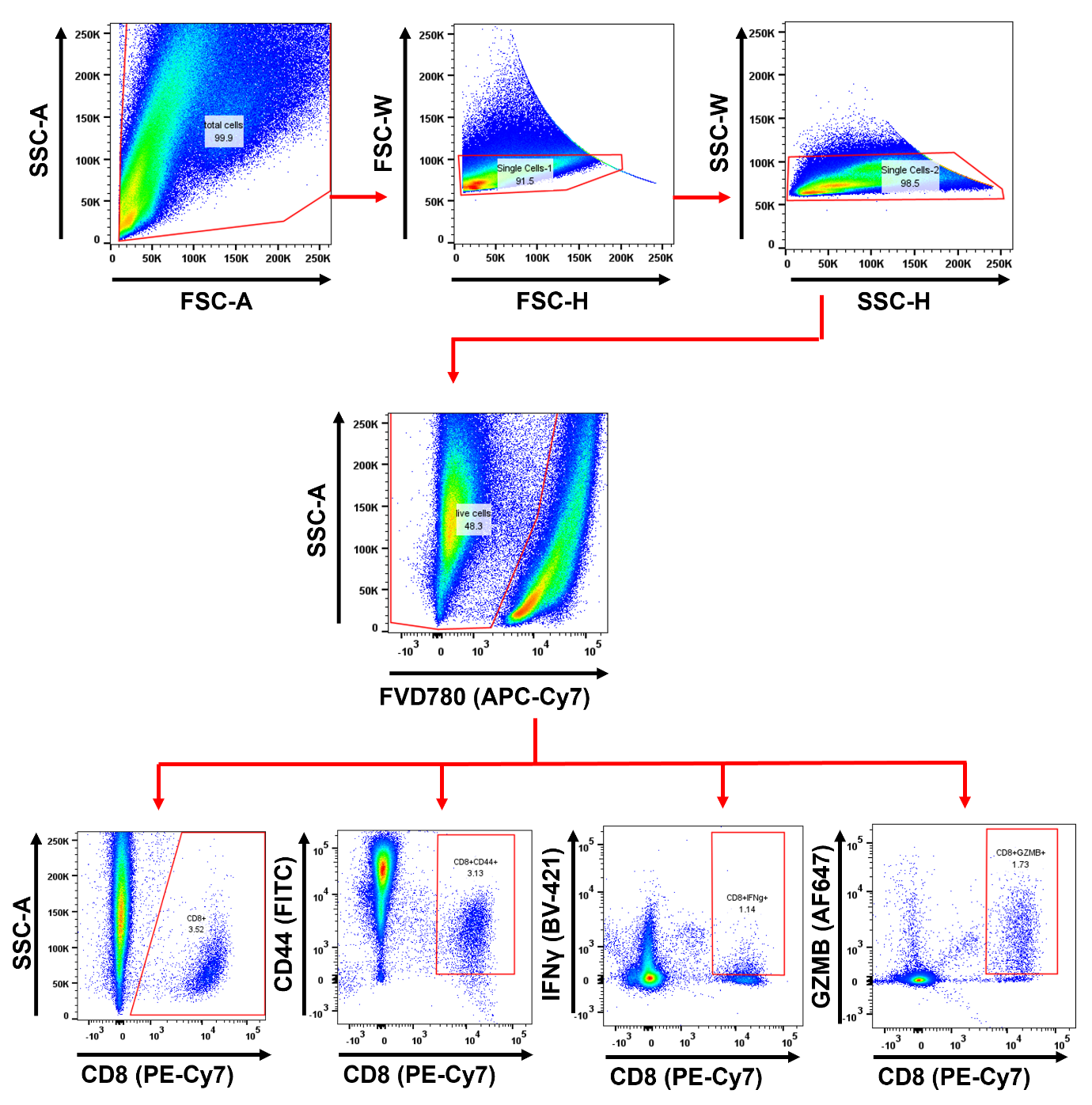
Supplementary Fig. S3**

**Fig. S3. A representative gating strategy for CTLs in tumor.**

To deplete dead cells, single cells in tumors were stained using Fixable Viability Dye eFluor 780 (FVD780). The live cells were stained using antibodies against CD8 to identify CTLs.

**Supplementary Fig. S4**

**
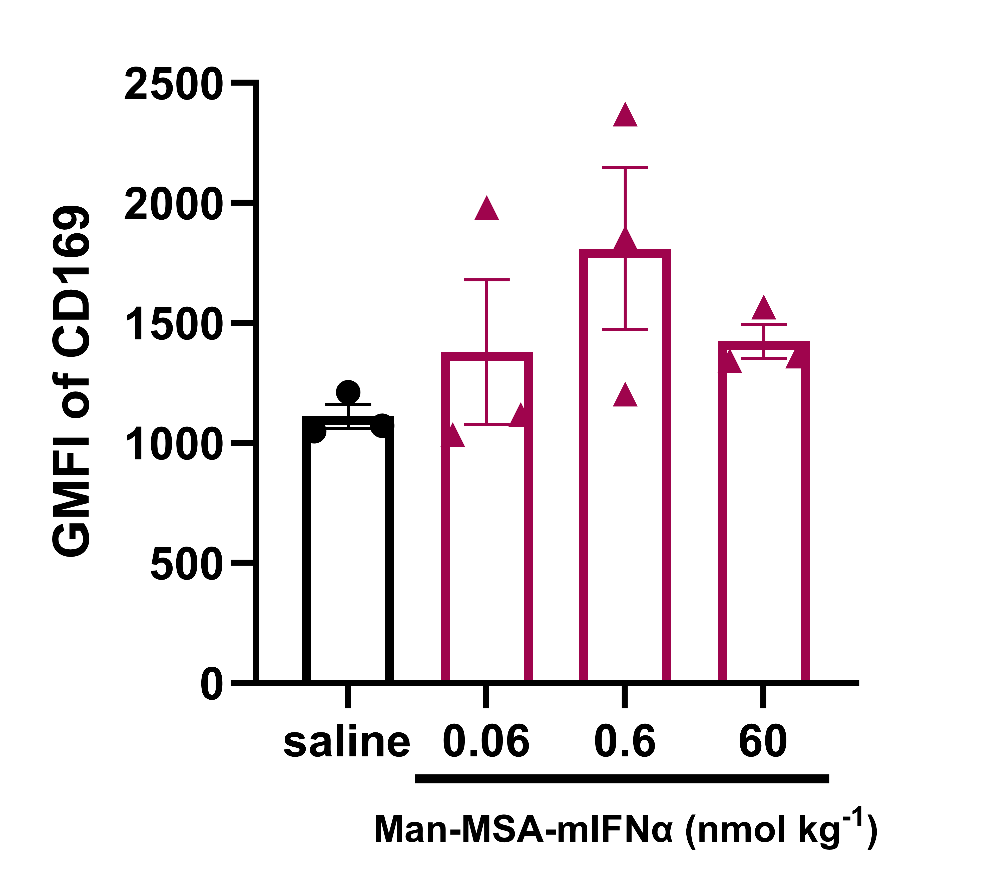
**

**Fig. S4. The optimal dose of Man-MSA-mIFNα for CD169 induction in vivo.**

C57BL/6N mice were subcutaneously administered Man-MSA-mIFNα (0.06, 0.6, 60 nmol kg^-1^) into the inguinal region. Thirty-six hours after administration, inguinal LNs were harvested and CD169 expression per cell evaluated in CD11b⁺ F4/80⁺ cells by flow cytometry (*n* = 3). Data are averages ± S.E. This experiment was performed once.

**
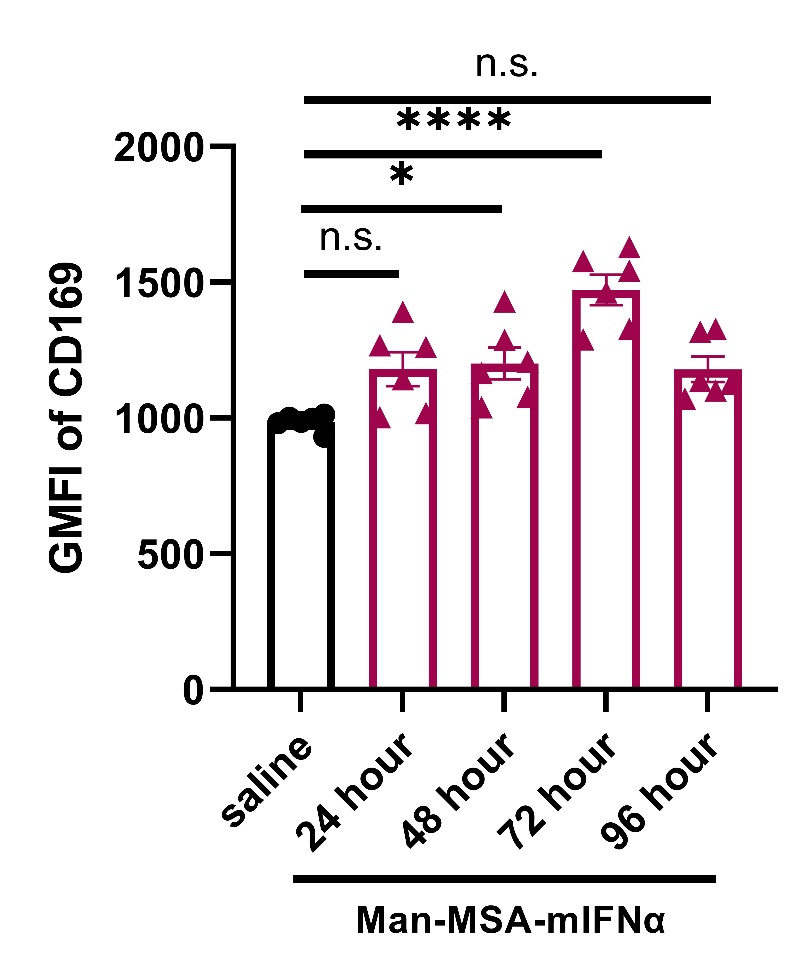
Supplementary Fig. S5**

**Fig. S5. The time course of CD169 expression in vivo.**

Man-MSA-mIFNα (0.6 nmol kg^-1^) was subcutaneously administered into the inguinal region of C57BL/6N mice. At each time point after administration, inguinal LNs were harvested and CD169 expression per cell evaluated in CD11b⁺ F4/80⁺ cells by flow cytometry (*n* = 6). Data are averages ± S.E. **p* < 0.05, *****p* < 0.0001, n.s.=nonsignificant. This result is representative of two independent experiments.

**
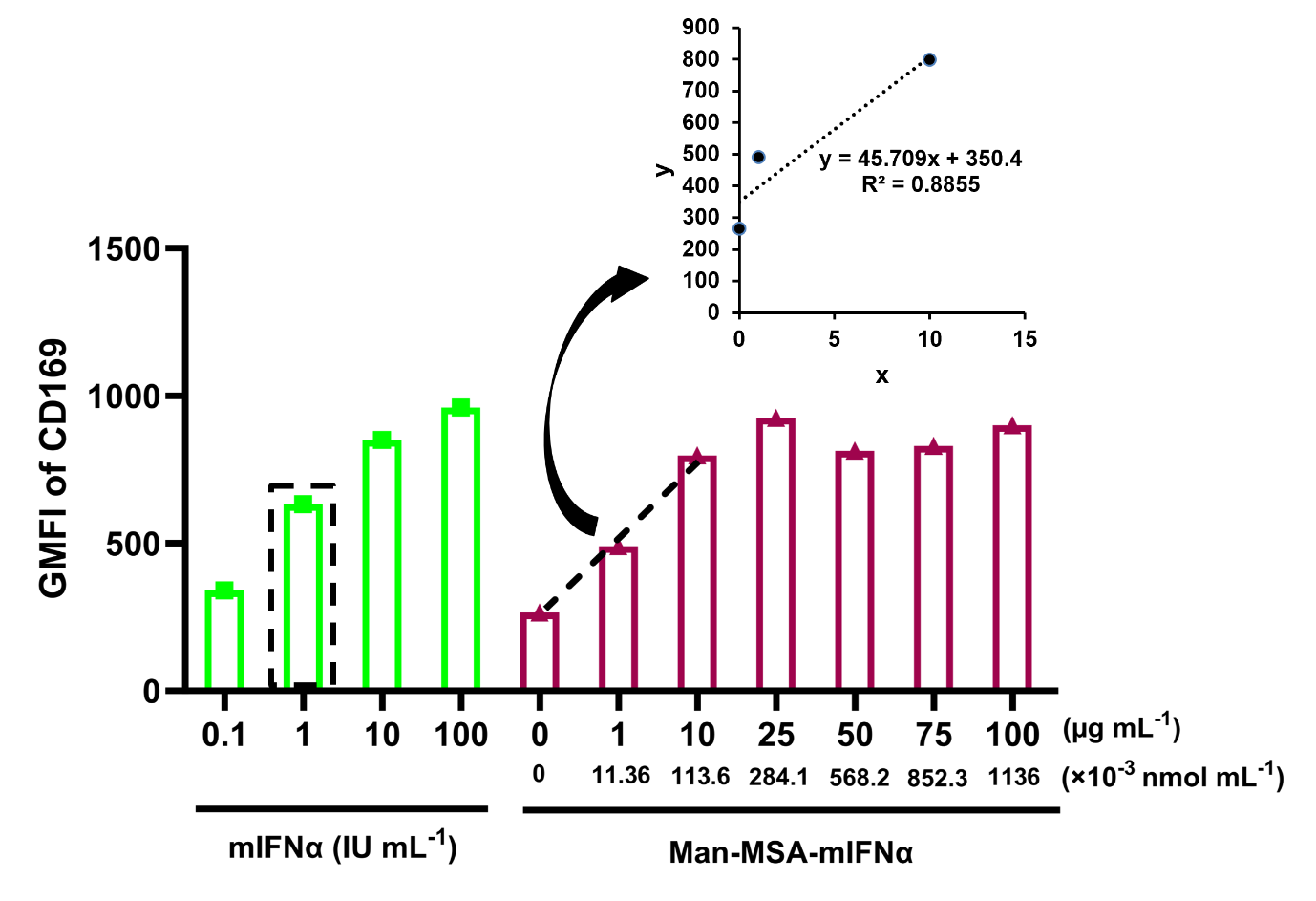
Supplementary Fig. S6**

**Fig. S6. The titer of mIFNα or Man-MSA-mIFNα for CD169 induction in vitro.**

J774.1 cells were incubated with each protein, followed by the evaluation of CD169 expression by flow cytometry. The amount of Man-MSA-mIFNα at the titer equal to 1 IU mL^−1^ mIFNα was estimated using a calibration curve of Man-MSA-mIFNα. This experiment was performed once.

**
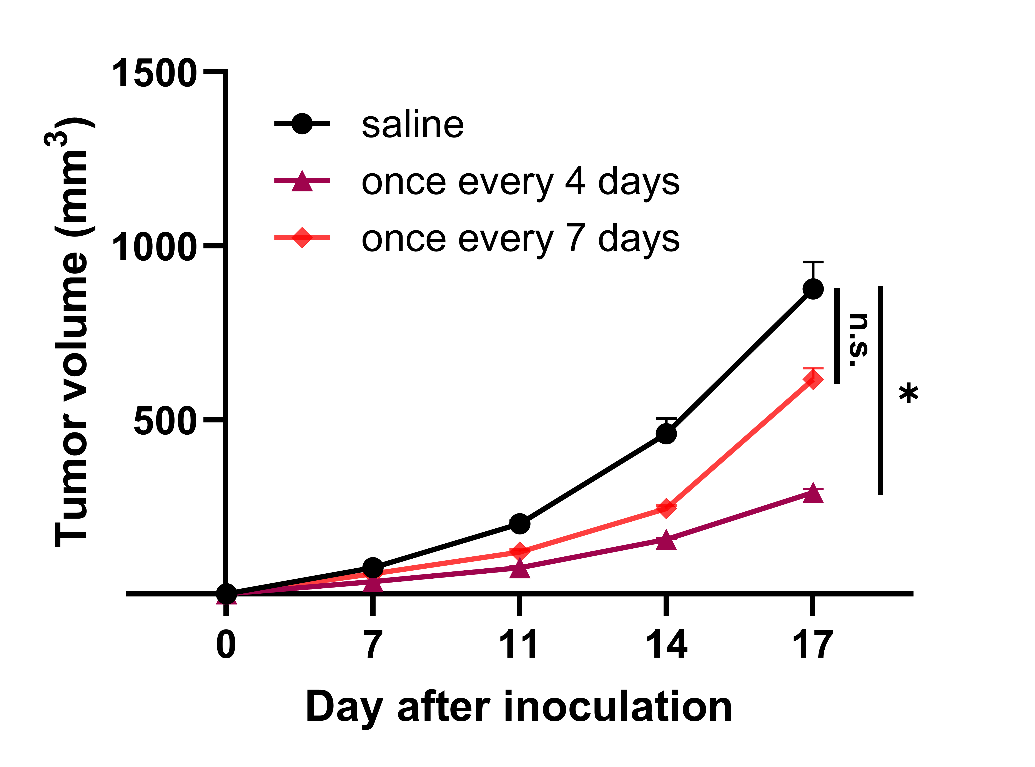
Supplementary Fig. S7**

**Fig. S7. Evaluation of the optimal dosing interval using MB49-bearing mice.**

Mice were treated subcutaneously with Man-MSA-mIFNα (0.6 nmol kg^-1^) near the tumor. Subcutaneous tumor volume was measured at each time point (*n* = 5–6). Data are averages ± S.E. **p* < 0.05, n.s.=nonsignificant. This result is representative of two independent experiments.

**
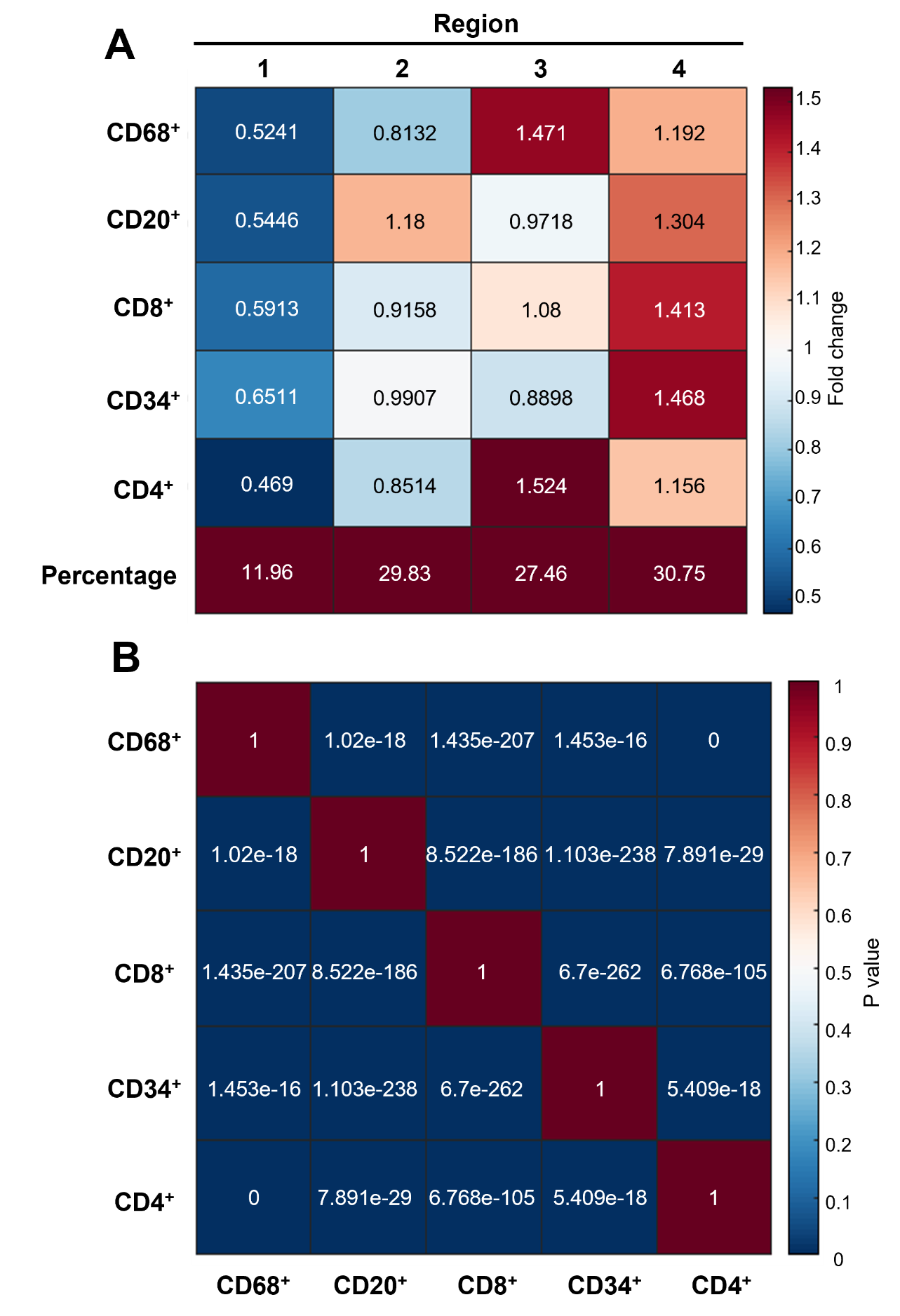
Supplementary Fig. S8**

**Fig. S8. (A) The cell composition of neighborhoods in CytoMAP (the number of cells per total cells in neighborhood) and (B) the P value of Pearson correlation coefficients.** These results are representative of two independent experiments.

**Supplementary Fig. S9**

**
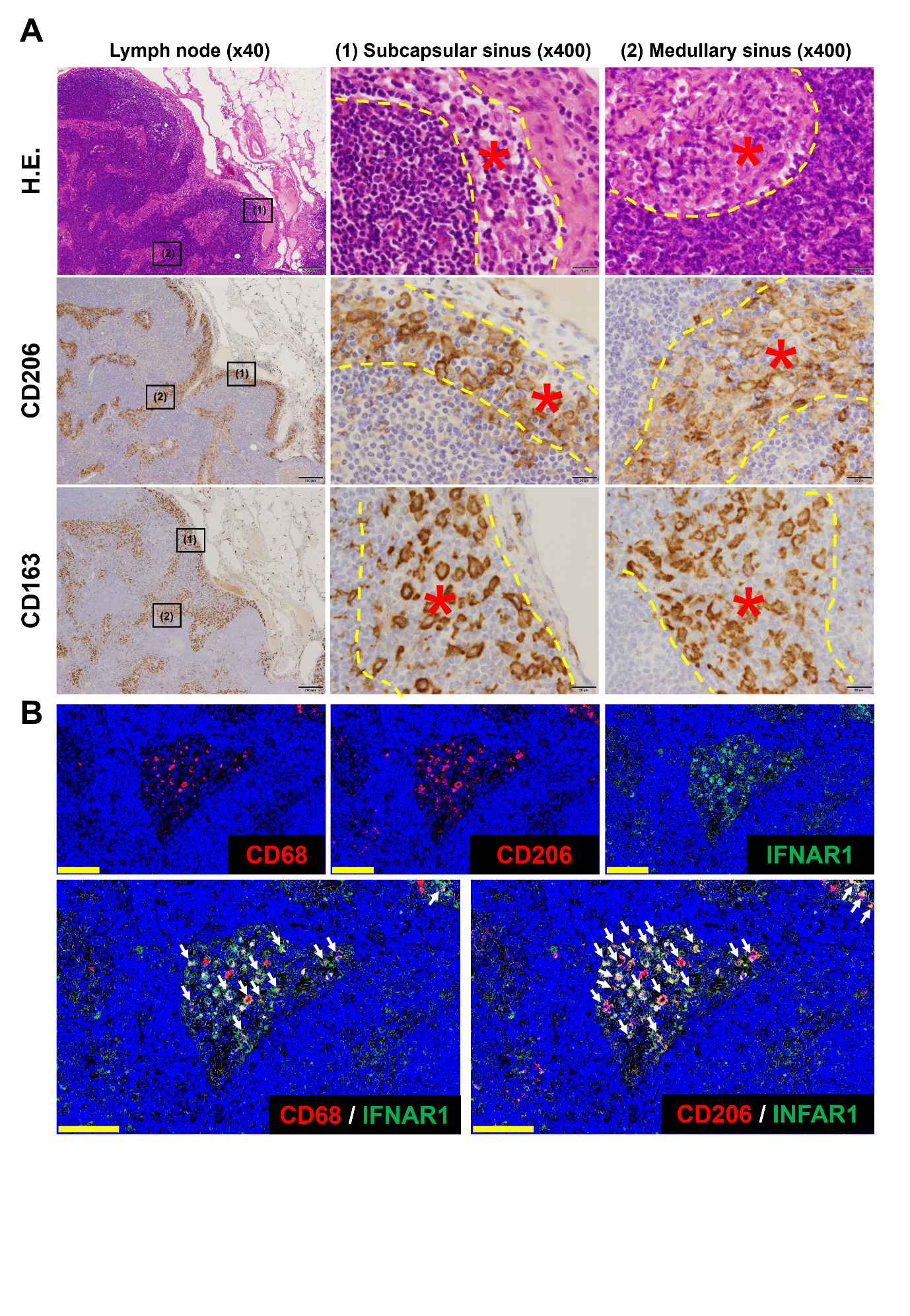
**

**Fig. S9. Expression of mannose receptor and type Ⅰ IFN receptor in LN macrophages.** A) H.E. staining and immunohistochemical analysis of CD206 and CD163 for regional LN of colorectal cancer patients. (*) indicates the sinus area. Scale bar, left: 200 µm, middle, right: 20 µm. B) Triple immunostaining with CD68 (red), CD206 (red), and IFNAR1 (green) in the sinuses of regional LNs of colorectal cancer patients. The white arrow indicates a double-positive cell. Scale bar: 100 µm. These results are representative of two independent experiments.

**
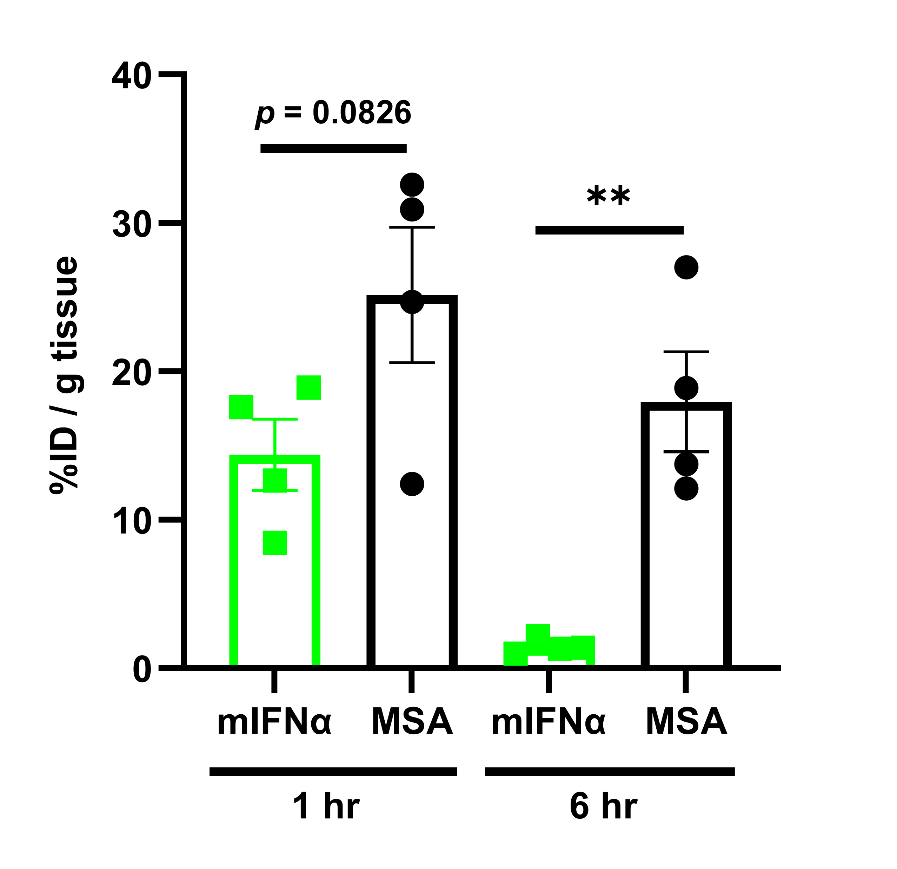
Supplementary Fig. S10**

**Fig. S10. Lymphatic drainage of albumin.**

Lymphatic drainage of albumin was measured 1 hour and 6 hours after subcutaneous (s.c.) administration of mIFNα and MSA (*n* = 4). Data are averages ± S.E. ***p* < 0.01. This experiment was performed once.

**
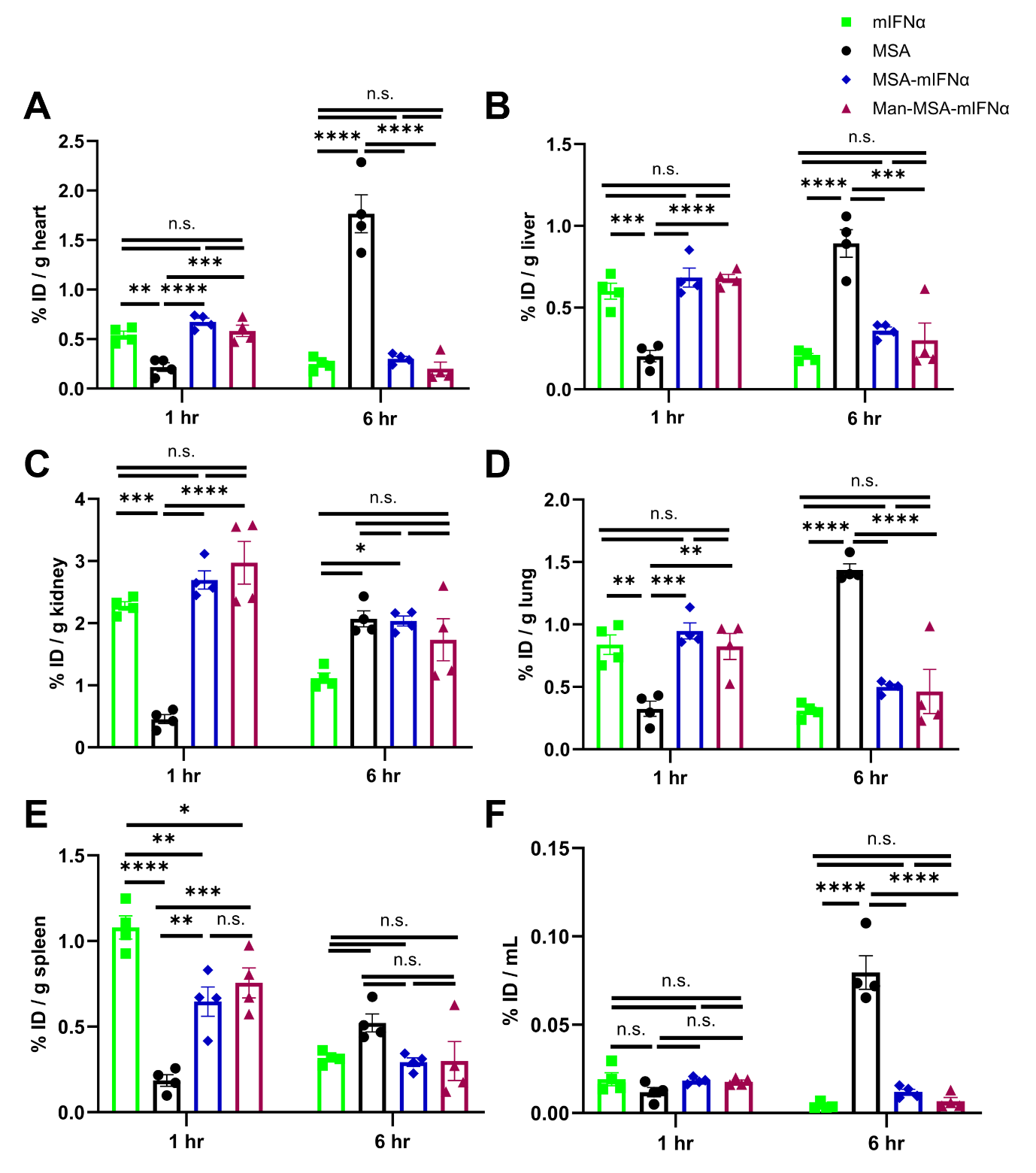
Supplementary Fig. S11**

**Fig. S11. Pharmacokinetic analysis of ^125^I-labeled Man-MSA-mIFNα.**

(A) Heart, (B) liver, (C) kidney, (D) lung, and (E) spleen distribution and (F) blood concentrations were measured 1 hour and 6 hours after administration (s.c.) of each protein (*n* = 4). Data are averages ± S.E. **p* < 0.05, ***p* < 0.01, ****p* < 0.001, *****p* < 0.0001, n.s.=nonsignificant. This experiment was performed once.

**Supplementary Fig. S12**

**
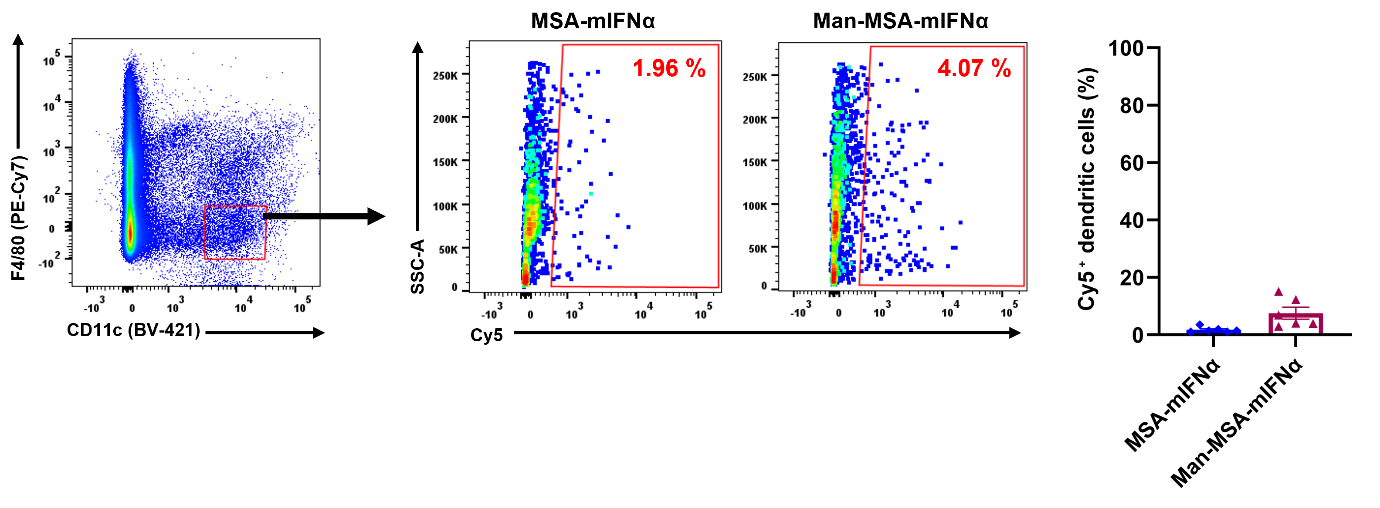
**

**Fig. S12. Distribution of Man-MSA-mIFNα to LN dendritic cells.**

The rate of Cy5-incorporated cells in CD11c^+^ F4/80^−^ dendritic cells 1 hour after subcutaneous administration was evaluated by flow cytometry (*n* = 6). Data are averages ± S.E. This result is representative of two independent experiments.

**
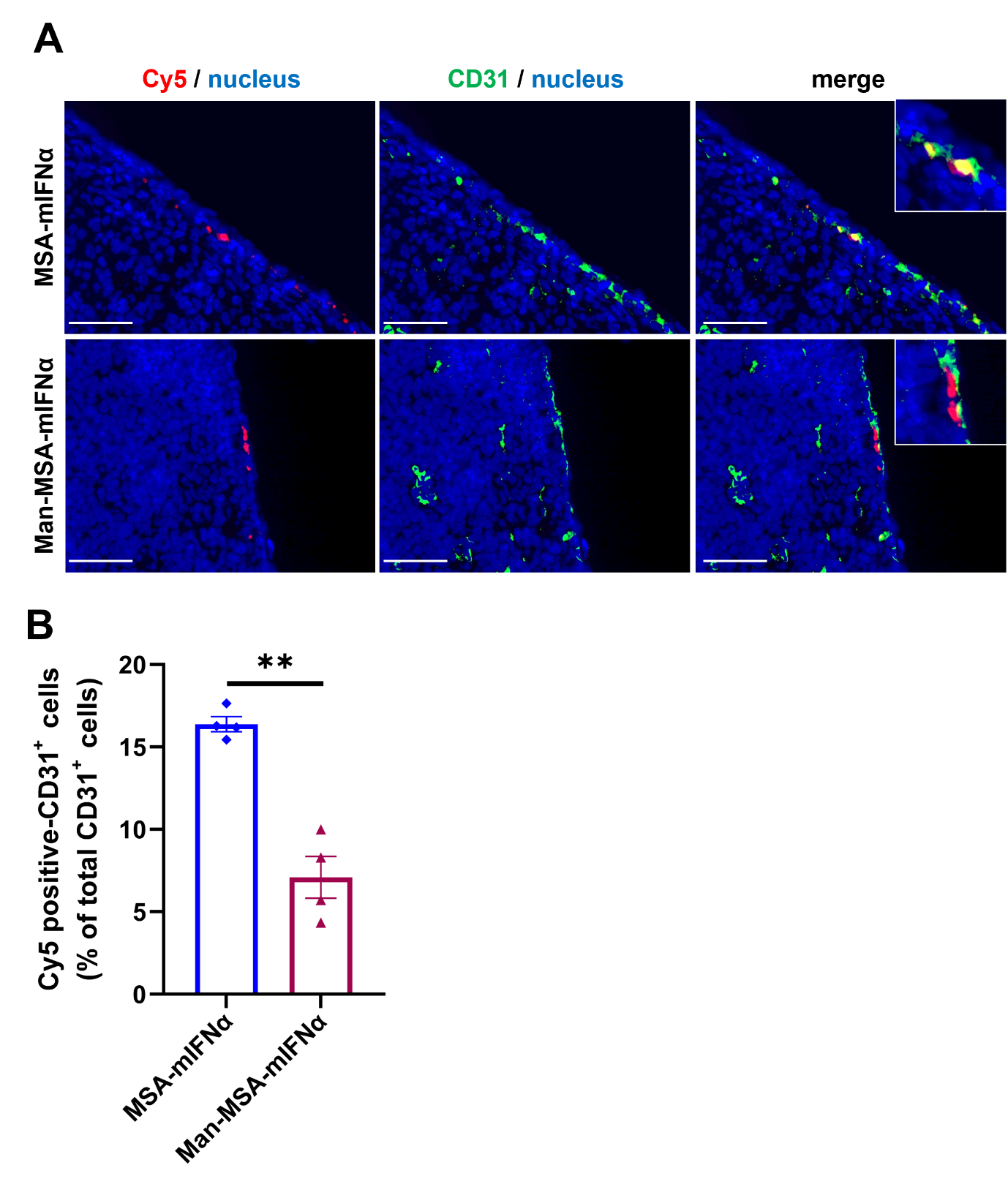
Supplementary Fig. S13**

**Fig. S13. IntraLN distribution of Man-MSA-mIFNα.**

(A) Representative images of immunofluorescence double staining for Cy5 and CD31. (B) Quantification of Cy5⁺ CD31⁺ cells (*n* = 4). Scale bar: 50 µm. Data are averages ± S.E. ***p* < 0.01. This result is representative of two independent experiments.

**
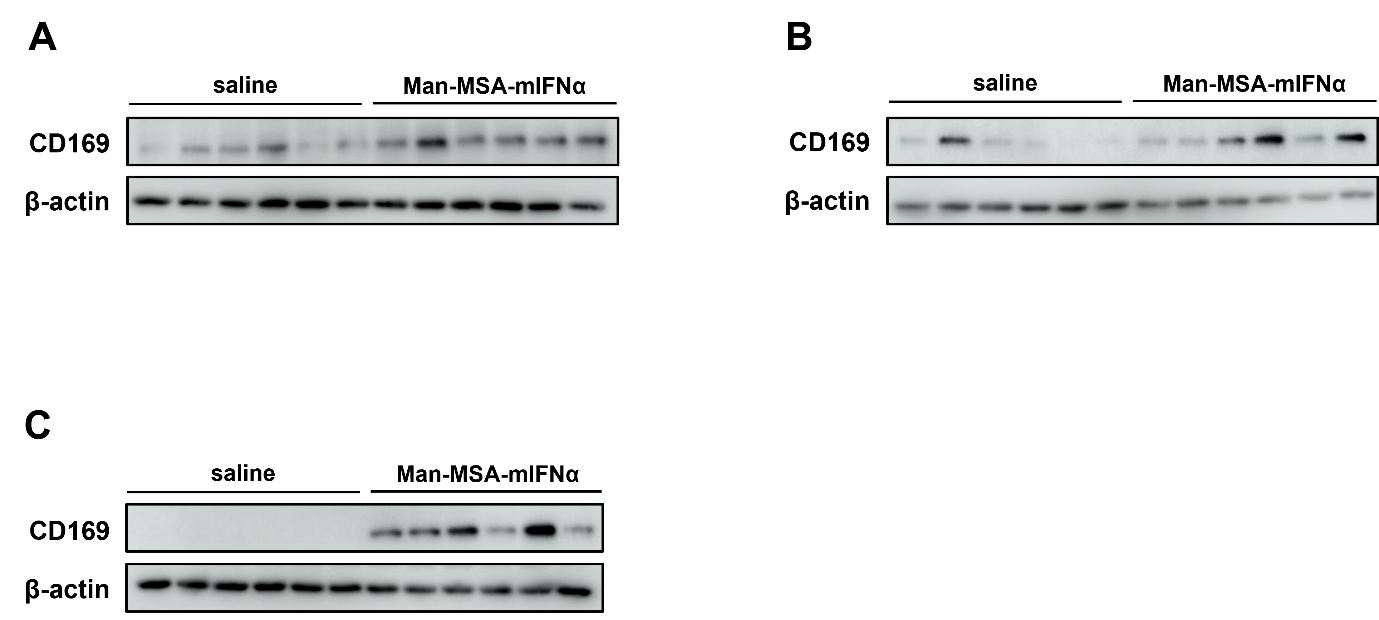
Supplementary Fig. S14**

**Fig. S14. CD169 induction by Man-MSA-mIFNα in tumor-draining LN.**

Western blotting analysis of CD169 expression in regional LNs of (A) MB49, (B) MC38 and (C) LLC-bearing mice. These results are representative of two independent experiments.

**
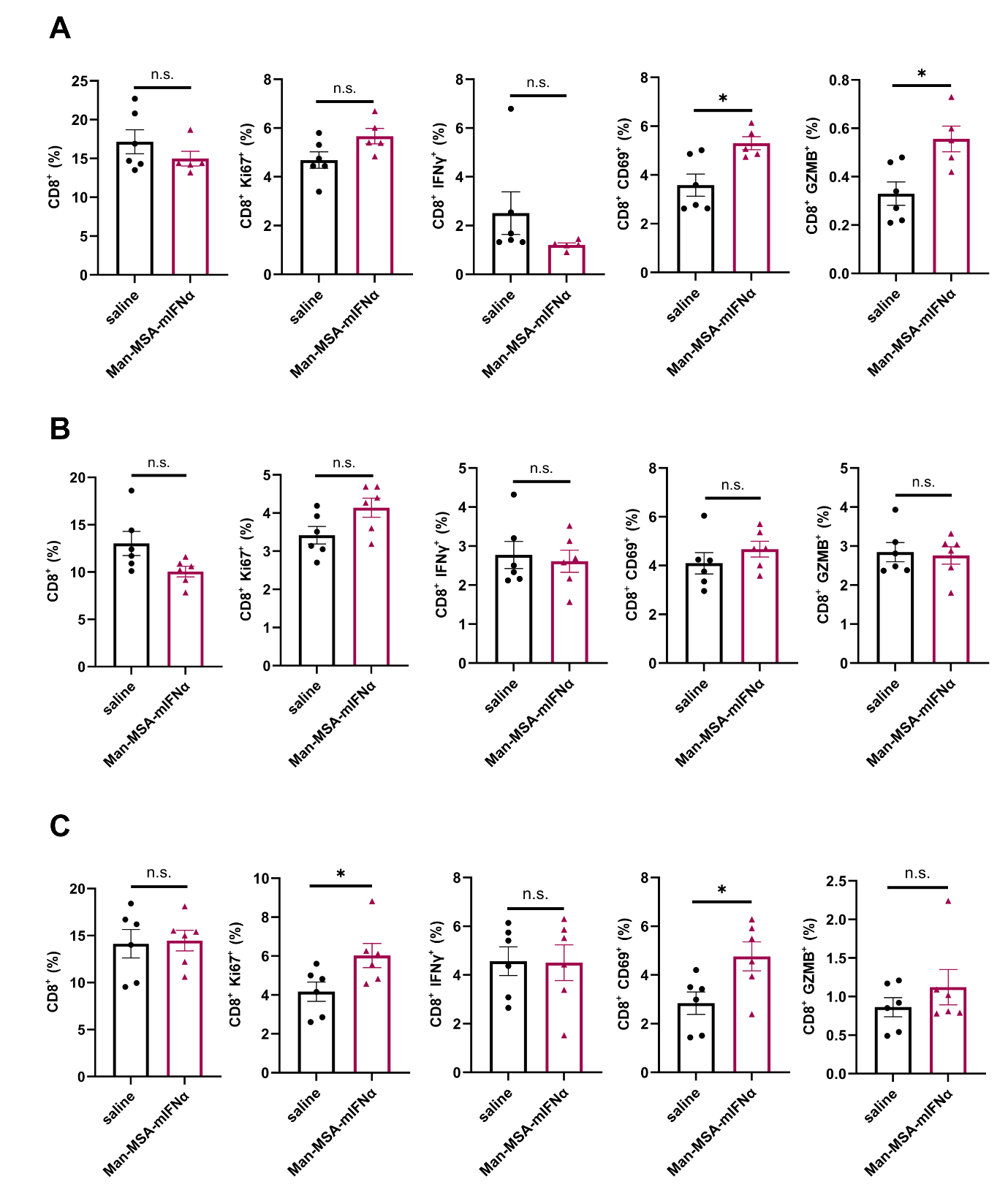
Supplementary Fig. S15**

**Fig. S15. Effect of Man-MSA-mIFNα on the phenotype of CD8⁺ T cells in LN.**

Inguinal LNs were harvested from (A) MB49, (B) MC38 or (C) LLC-bearing mice on the 10th day after tumor inoculation, followed by the evaluation of the percentage of CD8^+^ cells, Ki67^+^ CD8^+^ cells, IFNγ^+^ CD8^+^ cells, CD69^+^ CD8^+^ cells and GZMB^+^ CD8^+^ cells in live cells by flow cytometry ((A): *n* = 5–6; (B, C): *n* = 6). Data are averages ± S.E. **p* < 0.05, n.s.= nonsignificant. These results are representative of two independent experiments.

**Supplementary Fig. S16**

**
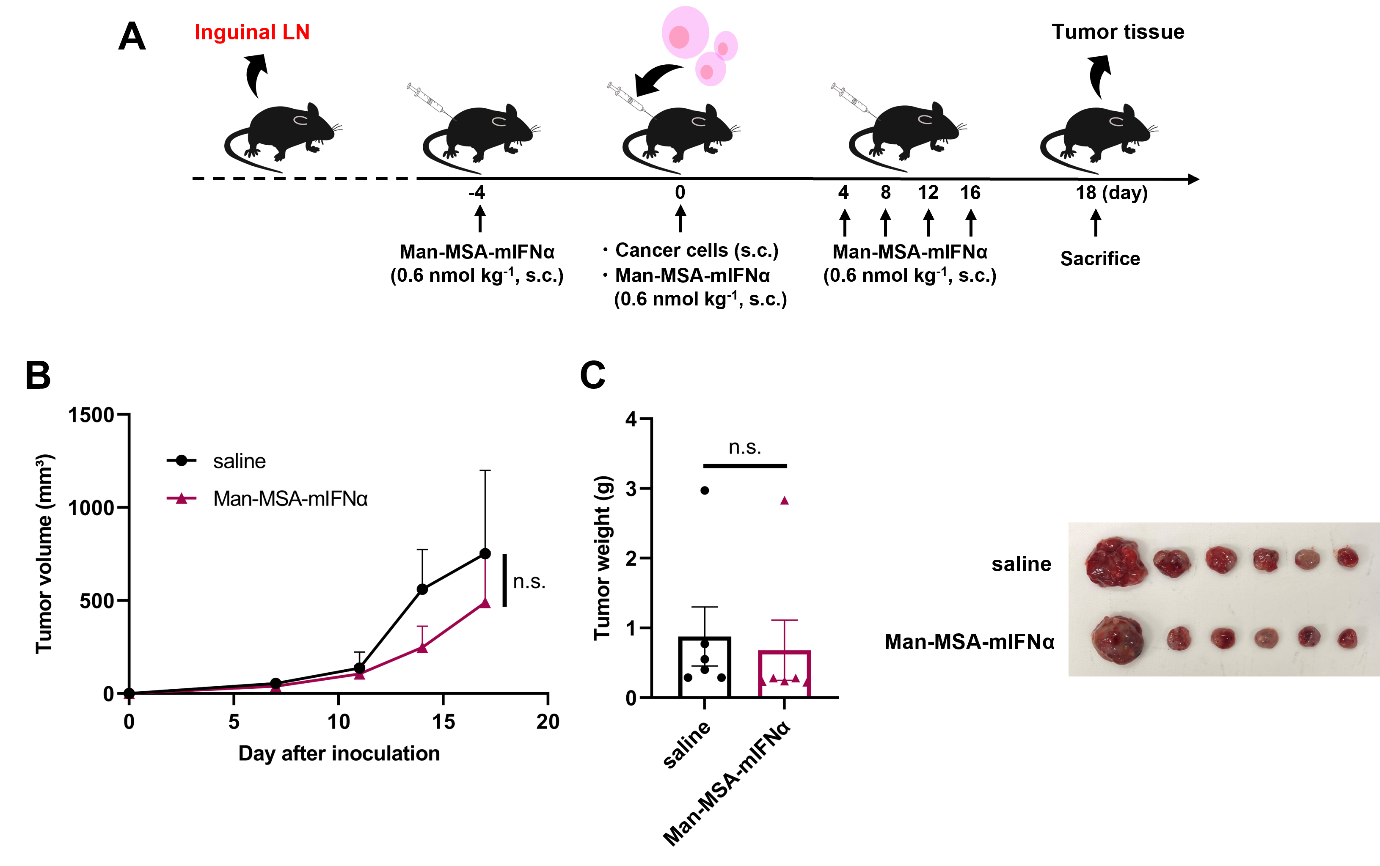
**

**Fig. S16. Effect of Man-MSA-mIFNα on tumor progression under the condition of removing the tumor-draining LN.**

A) The experimental protocol for the evaluation of the antitumor effect of Man-MSA-mIFNα in MB49-bearing mice after removing the tumor-draining LN. B) Subcutaneous tumor volume was measured in MB49-bearing mice at each time point (*n* = 6). C) Subcutaneous tumor weight (*n* = 6) (left) and the representative image of tumors (right) on the 18th day after tumor inoculation. Data are averages ± S.E. n.s.= nonsignificant. These results are representative of two independent experiments.

**
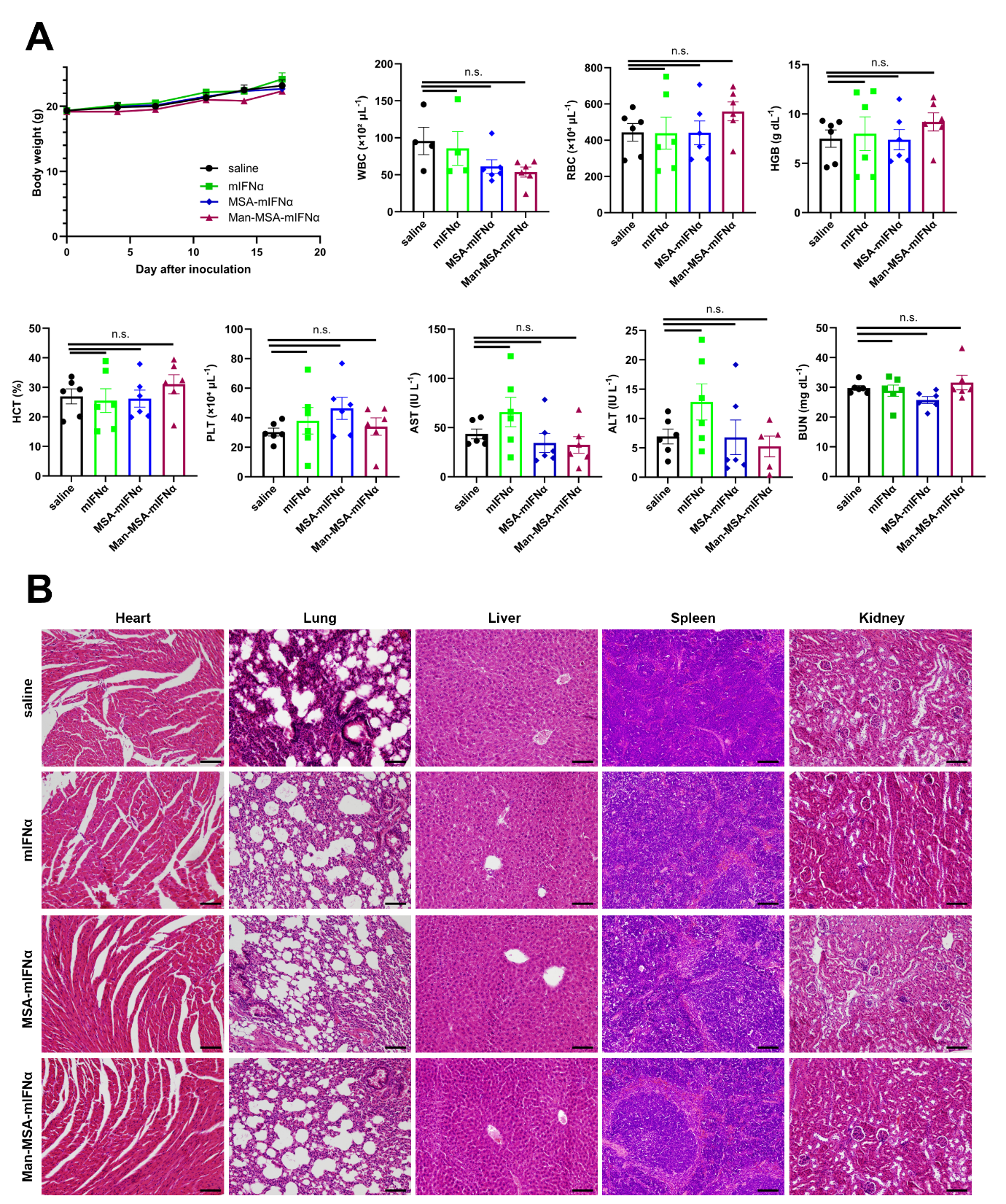
Supplementary Fig. S17**

**Fig. S17. Effect of Man-MSA-mIFNα on digestive symptoms, hematopoietic system, serum parameters and organ in MB49-bearing mice.**

A) The body weight was measured in MB49-bearing mice at each time point (*n* = 6). Blood parameters of blood cells (WBC, RBC, HGB, HCT, PLT), AST, ALT, and BUN in MB49-bearing mice on the 18th day after tumor inoculation (*n* = 4–6). B) Major organs were harvested from MB49-bearing mice on the 18th day after tumor inoculation, followed by H.E. staining. Scale bar: 100 µm. Data are averages ± S.E. n.s.=nonsignificant. These results are representative of two independent experiments.

**
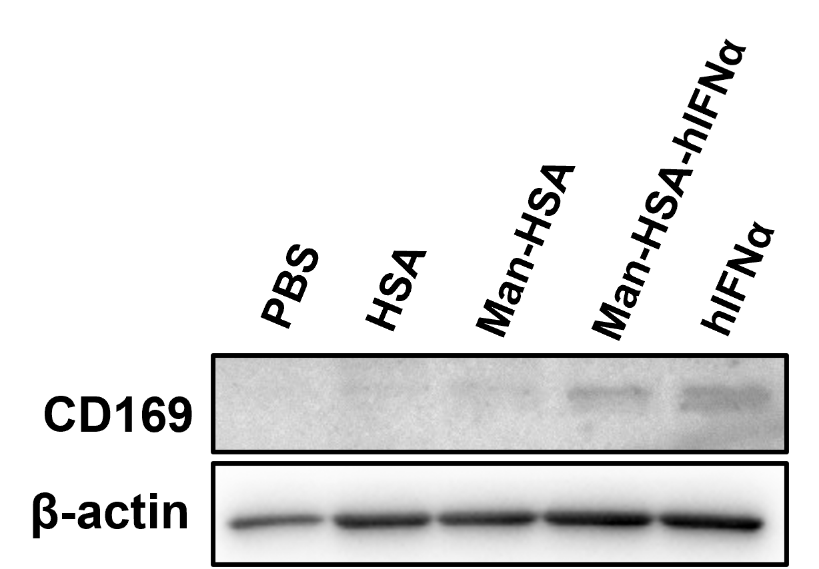
Supplementary Fig. S18**

**Fig. S18. CD169 induction by Man-HSA-hIFNα in human monocyte-derived macrophages.**

Human monocyte-derived macrophages were incubated with HSA (0.5 µM), Man-HSA (0.5 µM), Man-HSA-hIFNα (0.5 µM) and hIFNα (0.1 µg mL^-1^) for 24 hours at 37℃, followed by the evaluation of CD169 expression by western blotting. This result is representative of two independent experiments.

**Supplementary Table S1**. The list of antibodies for experiments.

| **Immunohistochemistry** |  |  |  |
| --- | --- | --- | --- |
| **Primary Antibody** | **Company** | **Catalog #** | **Dilution ratio** |
| CD68 | Dako | M0876 | 1:50 |
| CD169 | Abcam | ab183356 | 1:500 |
| CD206 | Abcam | ab64693 | 1:500 |
| CD163 | Novocastra | NCL-L-CD163 | 1:300 |
| CD8 | Cell Signaling Technology | 98941 | 1:400 |
| Ki67 | Dako | M7240 | 1:100 |
| **Secondary Antibody** | **Company** | **Catalog #** | **Dilution ratio** |
| Histofine Simple Stain MAX PO (M) | NICHIREI BIOSCIENCES INC. | 424132 | 1:1 |
| Histofine Simple Stain MAX PO (R) | NICHIREI BIOSCIENCES INC. | 424142 | 1:1 |
| **Western blotting (for physicochemical analysis)** |  |  |  |
| **Primary Antibody** | **Company** | **Catalog #** | **Dilution ratio** |
| MSA | Abcam | ab19195 | 1:2,000 |
| His-tag | Gene Tex | GTX115045 | 1:2,000 |
| **Secondary Antibody** | **Company** | **Catalog #** | **Dilution ratio** |
| HRP-conjugated rabbit anti-goat IgG | Santa Cruz | sc-2768 | 1:10,000 |
| HRP-conjugated mouse anti-rabbit IgG | Santa Curz | sc-2357 | 1:10,000 |
| **Western blotting (for assessment of CD169 expression)** |  |  |  |
| **Primary Antibody** | **Company** | **Catalog #** | **Dilution ratio** |
| CD169 | Bio-Rad | MCA947G | 1:2,000 |
| β-actin | Sigma-Aldrich | A5441 | 1:4,000 |
| **Secondary Antibody** | **Company** | **Catalog #** | **Dilution ratio** |
| HRP-conjugated goat anti-rat IgG | Proteintech | SA00001-15 | 1:10,000 |
| HRP-conjugated mouse anti-mouse IgG | Santa Curz | sc-516102 | 1:10,000 |
| **Immunofluorescence Staining** |  |  |  |
| **Primary Antibody** | **Company** | **Catalog #** | **Dilution ratio** |
| CD31 | Becton Dickinson | 550274 | 1:100 |
| **Secondary Antibody** | **Company** | **Catalog #** | **Dilution ratio** |
| Alexafluor 488 anti-rat IgG | Abcam | ab150157 | 1:200 |
| **Flow cytometry** |  |  |  |
| **Antibody (for cell surface marker)** | **Company** | **Catalog #** | **Dilution ratio** |
| BV-510-anti-CD11b | BioLegend | 562950 | 1:100 |
| PE-Cy7-anti-F4/80 | BioLegend | 123113 | 1:200 |
| PE-anti-CD169 | Invitrogen | 12-5755-82 | 1:100 |
| PE-Cy7-anti-CD8 | BioLegend | 100721 | 1:800 |
| PerCP-Cy5.5-anti-CD69 | BioLegend | 104521 | 1:100 |
| FITC-anti-CD44 | BioLegend | 103005 | 1:800 |
| BV-421-anti-CD11c | BioLegend | 117343 | 1:200 |
| **Antibody (for intracellular marker)** | **Company** | **Catalog #** | **Dilution ratio** |
| PE-anti-Ki67 | BioLegend | 652403 | 1:100 |
| BV-421-anti-IFN-γ | BioLegend | 505829 | 1:100 |
| AF647-anti-GZMB | Becton Dickinson | 560212 | 1:100 |
| **Multiplex IHC** |  |  |  |
| **Antibody** | **Company** | **Catalog #** | **Dilution ratio** |
| Akoya-Oligo CD68-BX015 | Akoya Biosciences | 232176 | 1:200 |
| Akoya-Oligo CD8a-BX029 | Akoya Biosciences | 232168 | 1:200 |
| Akoya-Oligo CD4-BX003 | Akoya Biosciences | 232174 | 1:200 |
| Akoya-Oligo CD20-BX007 | Akoya Biosciences | 232175 | 1:200 |
| Akoya-Oligo CD34-BX025 | Akoya Biosciences | 240076 | 1:200 |
| **Reporter** | **Company** | **Catalog #** | **Dilution ratio** |
| Alexa Fluor 647-RX015 | Akoya Biosciences | 6550027 | 1:50 |
| Atto 550-RX029 | Akoya Biosciences | 232059 | 1:50 |
| Cy5-RX003 | Akoya Biosciences | 6350001 | 1:50 |
| Alexa Fluor 750-RX007 | Akoya Biosciences | 232037 | 1:50 |
| Atto 550-RX025 | Akoya Biosciences | 6250019 | 1:50 |
| **Multiplex immunohistochemistry** | **Company** | **Catalog #** | **Concentration** |
| CD34 | Abcam | ab81289 | 1:200 |
| CD8 | Cell Signaling Technology | 98941 | 1:400 |
| CD4 | Cell Signaling Technology | 25229 | 1:400 |
| CD169 | Abbiotec | 251297 | 1:200 |
| B220 | Pharmingen | 553084 | 1:100 |
| CD68 | Dako | M0876 | 1:50 |
| CD206 | Abcam | ab64693 | 1:500 |
| IFNAR1 | Invitrogen | MA5-32006 | 1:200 |
| **In vivo depletion experiments** | **Company** | **Catalog #** | **Concentration** |
| Rat IgG2b isotype control-In Vivo | Selleck | A2116 | 100 µg/100 µL |
| Anti-mouse CD4-In Vivo | Selleck | A2101 | 100 µg/100 µL |
| Anti-mouse CD8α-In Vivo | Selleck | A2102 | 100 µg/100 µL |

**Supplementary Table S2**. Sequence of mutagenic primers for point mutations of MSA (D494N) or mIFNα2 (N78Q).

| Mutation | Forward | Reverse |
| --- | --- | --- |
| MSA (D494N) | ACATATGTTTCATTAACTGTCAGAG | CTCTGACAGTTAATGAAACATATGT |
| mIFNα2 (N78Q) | AGGAGGGTTGCCTGCCAAGCAGCAG | CTGCTGCTTGGCAGGCAACCCTCCT |
