# Supplementary figures and images for: Lymph node macrophage-targeted interferon alpha boosts anticancer immune responses by regulating CD169-positive phenotype of macrophages
Source: Mol Cancer. 2025 May 3;24:132. doi: 10.1186/s12943-025-02324-8 (PMC12049019; doi:10.1186/s12943-025-02324-8)

Fig. 2B

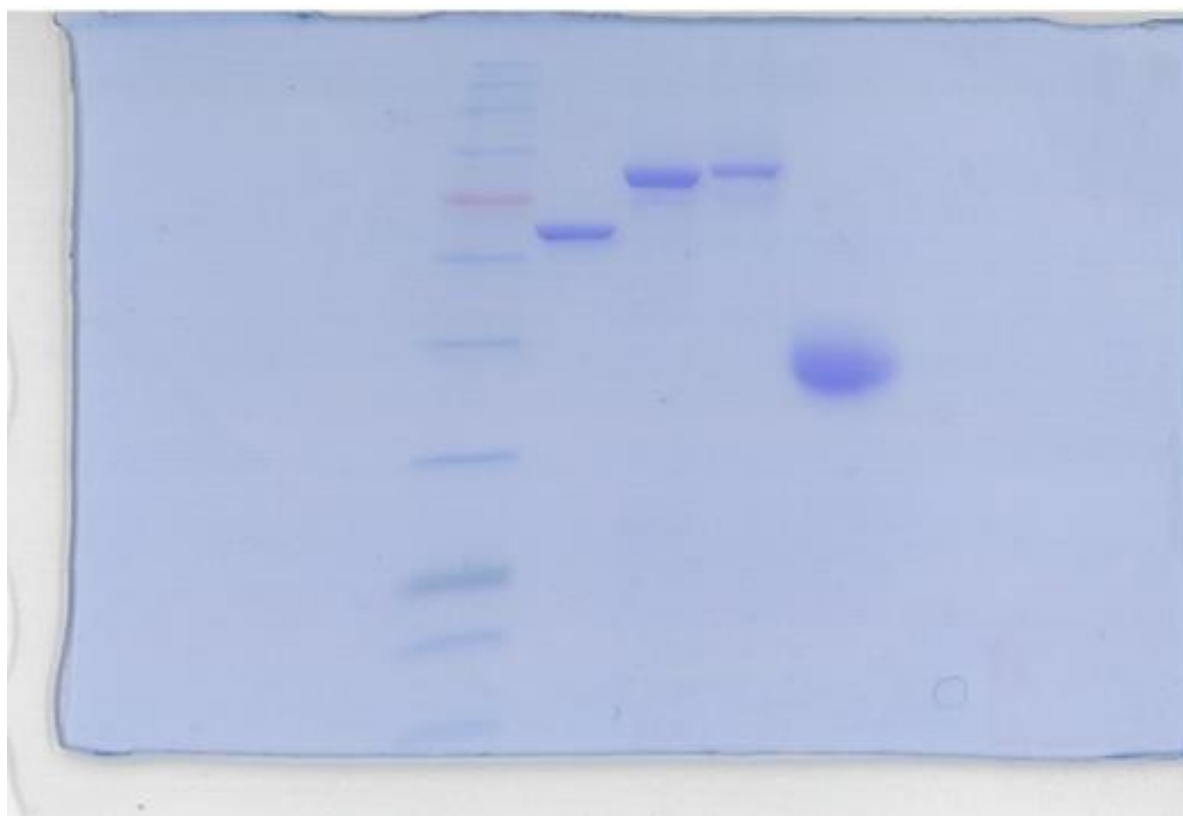

Fig. 2C

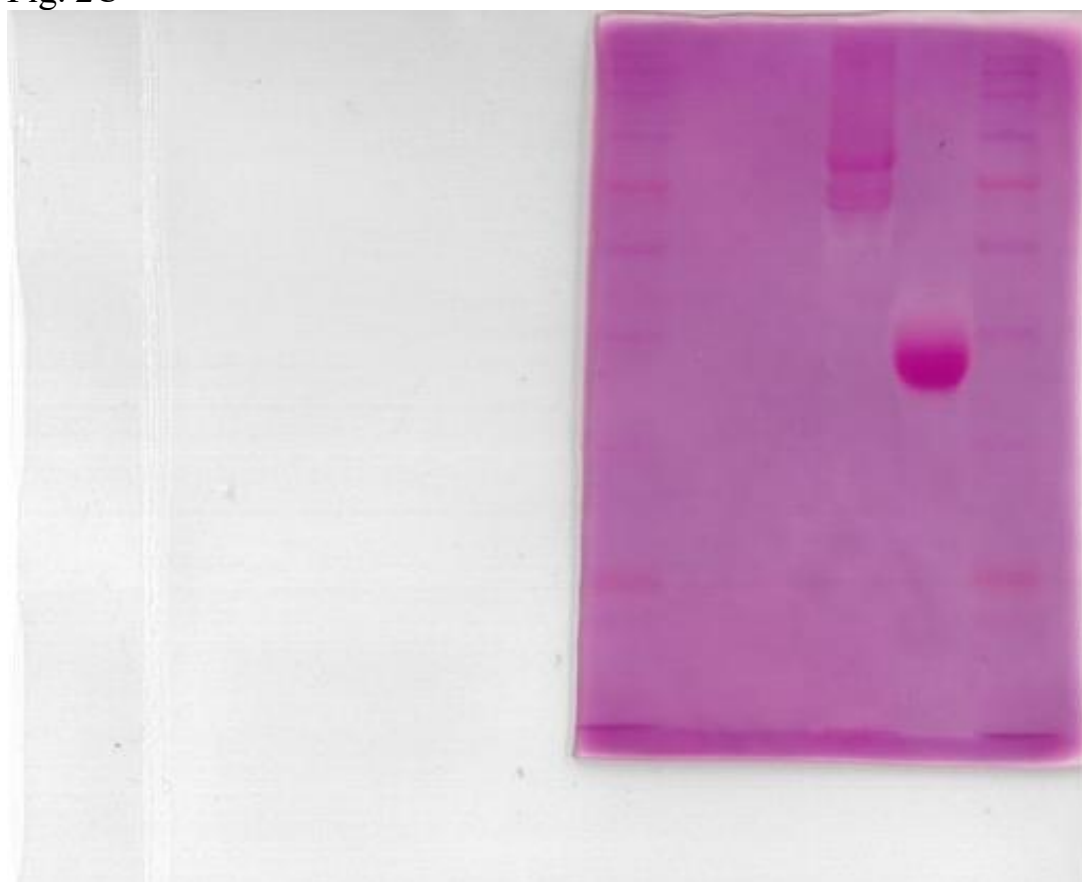

Fig. 2D

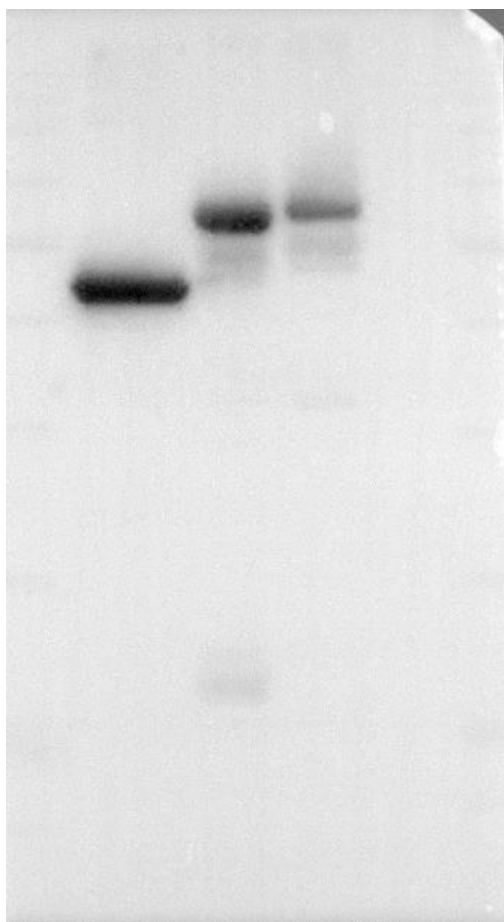

Fig. 2E

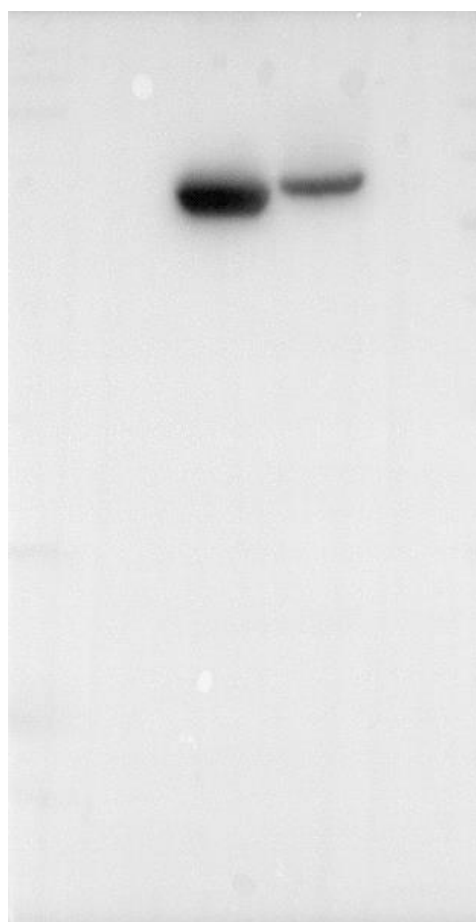

Fig. S14A

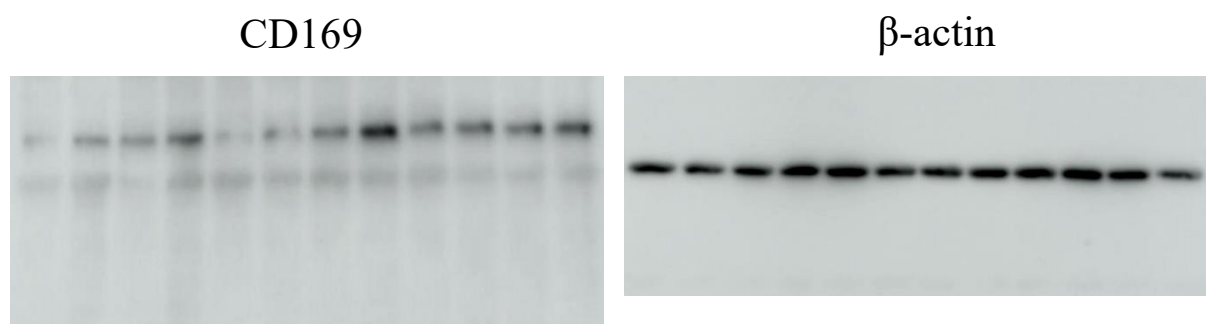

Fig. S14B

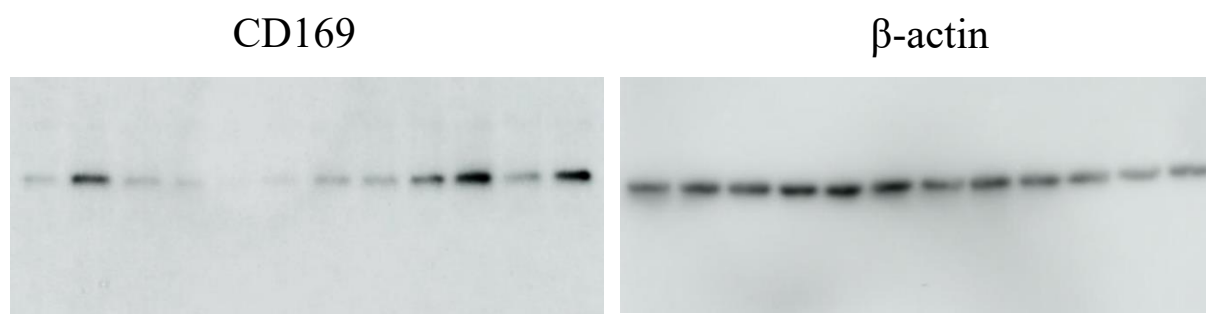

Fig. S14C

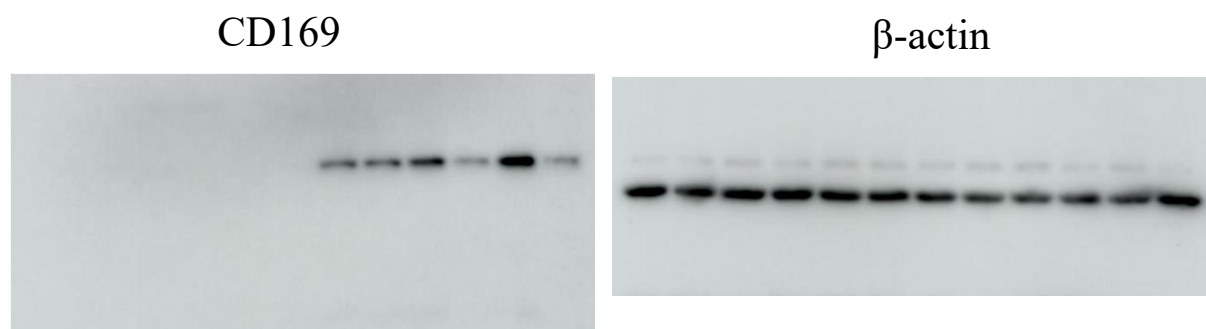

Fig. S18

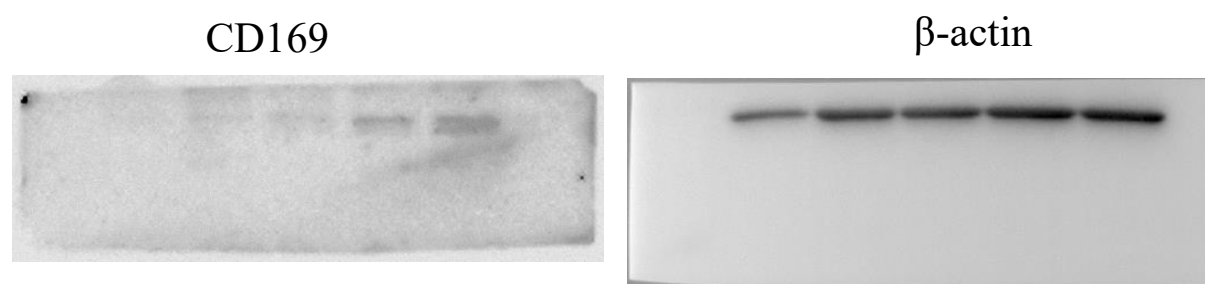

Supplement: Supplementary file 2 — Supplementary Material 2 [file 12943_2025_2324_MOESM2_ESM.pdf]
